# Exogenous α-ketoglutarate Modulates Redox Metabolism and Functions of Human Dendritic Cells, Altering Their Capacity to Polarise T Cell Response

**DOI:** 10.7150/ijbs.91109

**Published:** 2024-01-20

**Authors:** Marijana Milanović, Marina Bekić, Jelena Đokić, Dragana Vučević, Miodrag Čolić, Sergej Tomić

**Affiliations:** 1 Medical Faculty of the Military Medical Academy, University of Defense, Belgrade, Serbia.; 2 Department for Immunology and Immunoparasitology, Institute for the Application of Nuclear Energy, University of Belgrade, Belgrade, Serbia.; 3 Institute for Molecular Genetics and Genetical Engineering, University in Belgrade, Belgrade, Serbia.

**Keywords:** α-ketoglutarate, Dendritic cells, Th polarisation, redox metabolism, autophagy, regulatory T cells

## Abstract

Alpha-ketoglutarate (αKG) emerged as a key regulator of energetic and redox metabolism in cells, affecting the immune response in various conditions. However, it remained unclear how the exogenous αKG modulates the functions of dendritic cells (DCs), key cells regulating T-cell response. Here we found that non-toxic doses of αKG display anti-inflammatory properties in human APC-T cell interaction models. In a model of monocyte-derived (mo)DCs, αKG impaired the differentiation, and the maturation of moDCs induced with lipopolysaccharide (LPS)/interferon (IFN)-γ, and decreased their capacity to induce Th1 cells. However, αKG also promoted IL-1β secretion by mature moDCs, despite inflammasome downregulation, potentiating their Th17 polarizing capacity. αKG induced the expression of anti-oxidative enzymes and hypoxia-induced factor (HIF)-1α in moDCs, activated Akt/FoxO1 pathway and increased autophagy flux, oxidative phosphorylation (OXPHOS) and glycolysis. This correlated with a higher capacity of immature αKG-moDCs to induce Th2 cells, and conventional regulatory T cells in an indolamine-dioxygenase (IDO)-1-dependent manner. Additionally, αKG increased moDCs' capacity to induce non-conventional T regulatory (Tr)-1 and IL-10-producing CD8+T cells via up-regulated immunoglobulin-like transcript (ILT3) expression in OXPHOS-dependent manner. These results suggested that exogenous αKG-altered redox metabolism in moDCs contributed to their tolerogenic properties, which could be relevant for designing more efficient therapeutic approaches in DCs-mediated immunotherapies.

## Introduction

Alterations in the metabolism of immune cells are recognised as critical events shifting the immune response between homeostasis and inflammation 1. A great interest emerged in α-ketoglutarate (αKG, 2-oxoglutarate), an intermediate in the Krebs cycle with a complex and pleiotropic role in cell metabolism 2. αKG can act as a source of metabolic energy and amino acids 3 as well as a signalling molecule 4. Interestingly, myeloid cells, but not lymphoid immune cells, were found to express oxoglutarate receptor 1 (OXGR1, GPR99), mediating G-coupled intracellular signalling upon ligation 5, although it is not clear yet whether αKG is its physiological ligand 6. Within the cells, αKG is a substrate of the 2-oxoglutarate-dependent dioxygenases (OGDD), which critically regulate the stabilisation of hypoxia-inducible factor (HIF)-1α in various cells 1,4, and the expression of genes via regulation of DNA demethylases 7. Several papers suggested previously that the HIF-dependent mechanism of αKG could be utilised to inhibit tumour growth 8-10. However, the pro-tumorigenic effects of αKG were demonstrated as well 11, so it remained unclear whether αKG would be beneficial or adverse in tumour therapy. In addition, αKG was shown to regulate gene expression and cell signalling pathways related to growth and ageing, including the mammalian target of rapamycin (mTOR) and AMP-activated protein kinase (AMPK) 3. These activities were shown to extend the lifespan of several model organisms, including *Caenorhabditis elegans* 12, *Drosophila* sp. 13 and mice 14, and could reduce morbidity in humans 9. However, the increase in lifespan could also be linked to diminished resistance to infections, leading to poor quality of life 15. These studies emphasize the need for the evaluation of αKG effects on immune response and the underlying cellular and immunological mechanisms, considering that the immune response is critically involved in ageing, tumour progression, infections, and tissue homeostasis.

The regulation of immune response critically depends on the interaction between innate and adaptive immunity, particularly antigen-presenting cells (APC)-T cell interactions. Thereby, dendritic cells (DCs) are recognised as the most potent APC able to induce activation and differentiation of naïve T cells 16. DCs can sense danger signals via toll-like receptors (TLR) and cytokines receptors 17, and migrate to lymph nodes in a CCR7-dependent manner during maturation, increasing the expression of human leukocyte antigen (HLA)-DR, CD83, costimulatory molecules CD80, CD86, CD40, and produce high levels of innate pro-inflammatory cytokines (TNF-α, IL-1β, IL-6). Moreover, mature DCs secrete IL-12p70 which induces T helper (Th)1 cells and cytotoxic T lymphocytes (CTLs) which are necessary for an efficient anti-tumour response and resistance to intracellular infections 18. Alternatively, the combination of cytokines produced by DCs, such as IL-1β, IL-23, IL-6 and TGF-β, can promote the differentiation of Th17 18. The alteration of DC differentiation, such as with nanomaterials 19,20 or metabolism modulators 21,22, can promote their weak maturation and tolerogenic properties. In immature semi-matured and tolerogenic state, DCs display elevated expression of tolerogenic molecules such as Immunoglobulin-Like Transcripts (ILTs), Programmed Death-Ligand 1(PD-L1), Indoleamine 2,3-Dioxygenase (IDO)-1, and produce regulatory cytokines such as IL-10 and Transforming Growth Factor (TGF)-β 23. These molecules are critically involved in the induction of Th2 cells and different subsets of T regulatory cells (Tregs) which are necessary for tissue homeostasis and induction of tolerance 19,24. Thereby, enzymes involved in the regulation of the redox status in cells 17,25-27 and ROS 28,29, display detrimental effects on DC differentiation/maturation and functions. The same pathways are involved in autophagy in DCs, which may promote their tolerogenic properties 19. Although αKG was shown to be involved in various processes of redox metabolism and autophagy 3, its role in the regulation of DC functions has not been investigated thoroughly, particularly in humans.

Previous data suggested that dietary exogenous αKG can induce anti-inflammatory effects, via inhibition of inflammatory markers such as TLR4, nuclear factor (NF)-kB and mRNA for TNF-α in intestinal cells 30. Also, αKG inhibited lipopolysaccharide (LPS)-induced differentiation of M1 macrophages, and potentiated M2 differentiation via metabolic and epigenetic reprogramming of these cells 31,32. In contrast, αKG was shown to inhibit Treg differentiation and potentiated Th1 cells 33, and increased LPS-induced expression of CD86 and MHC class II in mouse bone-marrow-derived DCs 34, which could be interpreted as pro-inflammatory effects of αKG. Therefore, it remained unclear what are the immunological effects of exogenous αKG, particularly in DCs-mediated regulation of T cell response. Moreover, most previous studies used predominantly αKG analogues 31-33 or microparticles 34, presuming that the soluble form of αKG is cell impermeable. However, this view was disproven by the data showing that αKG analogues rapidly hydrolyse extracellularly and enter into different cells, thereby inducing αKG-dependent, but also analogue-dependent effects on cellular metabolism 35. Therefore, the primary aim of this study was to investigate the immunological mechanisms the of non-esterified form of αKG in models of human APC/T cell interaction. We hypothesised that the exogenous αKG can alter redox metabolism in moDCs, thereby modulating their differentiation, maturation, and functional capacity to polarise T-cell response.

## Materials and Methods

### Cells

The cytotoxicity and immunomodulatory properties of αKG were tested *in vitro* using models of human peripheral blood mononuclear cells (PBMCs) and co-cultures of monocyte-derived (mo) DCs and T cells, both isolated from PBMCs. Peripheral blood was collected from healthy donors, who provided written Informed consent before blood sampling, and all experiments were carried out in accordance with the Declaration of Helsinki after approval by the Ethical Committee of the Institute for the Application of Nuclear Energy (INEP). PBMCs were isolated from Na-EDTA-filled vacutainer tubes (Beckton Dickenson, New Jersey, USA) or buffy coats by density gradient centrifugation (Axis-Shield PoC AS, Oslo, Norway), according to the manufacturer's instructions. Monocytes and total T cells, or CD4^+^ T cells, were purified from PBMCs by negative magnetic-activated cell sorting (MACS) using Pan Monocyte Isolation Kit, Pan T cell Isolation Kit or CD4^+^ T cell isolation kit (all from Miltenyi Biotec, Bergisch Gladbach, Germany), according to manufacturer's instructions, thus providing >87% of purified CD14^+^ monocytes, and >95% of CD3^+^ T cells or CD4^+^ T cells, as detected by flow cytometry (LSR II, Beckton Dickenson, California, United States) (data not shown).

### Experimental design

#### Peripheral blood mononuclear cells

Freshly isolated PBMCs (3x10^5^/well of the 96-wells plate) were cultivated in a complete RPMI-1640 medium without L-glutamine, containing 10% foetal calf serum (FCS), 50 µM 2-mercaptoethanol (all from Sigma-Aldrich, St. Louis, Missouri, USA), and antibiotics (Penicillin and Streptomycin, 100U/ml, Merk Sigma). α-Ketoglutaric acid disodium salt dihydrate (α-KG, Millipore Sigma, Sigma-Aldrich Chemie GmbH, Taufkirchen, Germany) stock solution (1M) was prepared fresh in Dulbecco Modified Eagle Medium without L-glutamine (DMEM, Pan Biotech Germany) before each experiment, and the pH was set to 7.3 using NaOH and HCl before use in cultures. PBMCs were treated with different doses of αKG (1.2 mM - 200 mM) and incubated for the next 24-48h at 37℃, 5% CO_2_ and 90% humidity, followed by measurements of metabolic activity, cell death and ROS.

In addition, PBMCs were treated with phytohemagglutinin (PHA, Merk Sigma) at 20 µg/ml, and cultivated in the presence of αKG (10 mM, 25 mM and 50 mM) or its absence for the next 72h. The cell-free supernatants were collected and stored at -20^o^C for cytokines analyses. Similar PBMC cultures were set for measurements of MTT and cell proliferation. The proliferation of PBMCs was determined in [^3^H]-thymidine assay, as described previously 36. Briefly, the cells were pulsed with [^3^H]-thymidine for the last 18h of cultures (1 μCi/well; Amersham, Amersham, UK), and then harvested onto glass fibre filters, followed by drying at 50^o^C and dissolving in scintillation solution for β-particles counting in LKB-1219 (Rackbeta, Turku, Finland).

#### Monocyte-derived dendritic cells

To generate moDCs, MACS-purified monocytes (up to 1x10^6^ / mL) were cultivated in L-glutamine-free DMEM supplemented with 20 ng/ml of human recombinant granulocyte macrophages colony-stimulating factor (GM-CSF; Novartis, Basel, Switzerland) and 20 ng/ml of human recombinant IL-4 (Roche Diagnostics, Basel, Switzerland) for 4 days, to induce immature moDCs. The maturation of moDCs was induced by treating immature moDCs with 200 ng/ml of LPS (Escherichia coli 0.111:B4, Merk Sigma) and 20 ng/ml of human recombinant interferon (IFN)-γ (R&D Systems, Minneapolis, MN, United States) for next 16-18h. The effects of αKG on moDCs were tested by treating monocytes with αKG (10 mM or 50 mM) on day 0. One-quarter of the medium containing the equivalent amounts of GM-CSF, IL-4 and αKG, was refreshed on day 3 of the cultures. The control moDCs were differentiated likewise but without α-KG. In some experiments, additional moDCs cultures were treated with N-acetylcysteine (NAC, Merk Sigma, 10 mM), a mixture of Rotenone (Merk Sigma, 0.5 µM) and Antimycin A (0.5 µM), or their combination, on day 2 of the cultures. After the 5-day cultures, moDCs were harvested by light pipetting and washed twice, to remove the excess of free stimuli. moDCs number and viability were determined by using Trypan-blue viability dye and Muse cell count viability kit (Luminex Corporation, Texas, United States), and the analyses of phenotype, proteins and genes expression, ROS, autophagy, OXPHOS and glycolysis were determined, whereas the functions of moDCs were analysed in co-cultures with MACS-purified allogeneic T cells. Cell-free supernatants were collected and stored at -20 ℃ to detect cytokine levels.

#### Mixed leukocyte reaction

Allogeneic proliferation and Th polarisation were tested in co-cultures of moDCs with MACS-purified CD3^+^ or CD4^+^ T cells. MoDCs (1x10^4^ - 0.25x10^4^/ well of the round-bottom 96-wells plate) were co-cultivated with CellTrace Far Red (CTFR, Invitrogen)-labelled T cells (1x10^5^/well), at 1:10 -1:80 MoDC: T cell ratios, respectively. After 4 days of culture, CTFR dilution was determined by flow cytometry, after the exclusion of doublets and PI^+^ (dead cells).

For the T cell polarisation assay, moDCs/T cell co-cultures were carried out in 1:20 moDC: T cell ratios for 5 days, and then treated with Phorbol 12-myristate 13-acetate (PMA, 20 ng/mL) and ionomycin (500 ng/mL) (both from Merk Sigma) for the last 6h, before harvesting the cell-free supernatants for cytokines quantification. To detect intracellular cytokines in T cells, the same co-cultures were treated with PMA/ionomycin and monensin (2 μM, Sigma-Aldrich) for the last 4 h, and then prepared for the flow cytometry analysis. For the induction of regulatory T cells, moDC/T cell co-cultures were carried out in suboptimal (1:50) moDC: T cell ratio in the presence of a low dose of human recombinant IL-2 (2 ng/ml, R&D Systems) for 6 days. In some experiments these co-cultures were treated with indolamine dioxygenase (IDO)-1 inhibitor, 1-methyl-tryptophan (1-MT, 0.3 mM; Sigma-Aldrich Co.), blocking anti-ILT-3 Ab (2 μg/mL; R&D Systems) or isotype control Ab (anti-rat IgG2b; Thermo Fisher Scientific).

### Cytotoxicity assays, ROS and autophagy

The metabolic activity of PBMCs cultivated with α-KG was analysed after 24h and 48h in an MTT assay. MTT (3-(4,5-dimethylthiazol-2-yl)-2,5-diphenyltetrazolium bromide, Merk Sigma) at the final concentration of 0.5 mg/mL, was performed as described previously 24.

Apoptosis and necrosis of PBMCs were analysed by staining the permeabilised and non-permeabilised cells, with propidium iodide (PI, 50 μg/mL, Sigma-Aldrich), respectively, and the analyses by flow cytometry. The total % of dead (predominantly necrotic) cells was determined as the % of PI^+^ cells, whereas the % of apoptotic cells was determined by analysing sub-G1 peak in the samples that were fixed/permeabilised with ice-cold 75% ethanol, as described previously 24.

Reactive oxygen species (ROS) in PBMCs and moDCs were measured by using Muse Oxidative Stress Kit (Luminex Corporation, Texas, United States) containing dihydroethidium (DHE) dye as a probe and analysis on Guava Muse Cell Analyzer (Luminex). The autophagy flux was analysed in moDCs after the cultures, by using the Muse Autophagy Kit (Luminex), which is based on the detection of a membrane-converted variant of LC3 (LC3II). The moDCs were either stained directly or washed first in PBS and then incubated for 4 h in PBS in the presence of Bafilomycin, according to the manufacturer's protocol. The total expression of LC3-II was determined, and the autophagy flux was calculated as the ratio of bafilomycin-treated and non-treated cells in each experimental group.

### Oxygen consumption and extracellular acidification rates

Oxygen consumption rate (OCR, O_2_ mpH/min), as a measure of OXPHOS, and extracellular acidification rate (ECAR, mpH/h) were determined in moDCs collected after 5 days of cultivation, by using MitoXpress Xtra and pH-Xtra kits (both from Agilent Technologies, Santa Clara, CA, USA), respectively. For OCR measurements, moDCs were washed in serum-free DMEM and then seeded in triplicates (5 x 10^4^ cells/well) in flat-bottom Costar® 96-Well Black Polystyrene Plates for 1h, in DMEM containing 25 mM glucose, 1 mM pyruvate and 2 mM L-glutamine. In addition to basal OCR, each moDCs culture was treated with 2 μM oligomycin to inhibit mitochondrial complex V, 0.5 μM FCCP [0.5 mM carbonyl cyanide 4-(trifluoromethoxy) phenylhydrazone] to induce maximal respiration, or with 1 μM Rotenone and 1 μM Antimycin A (all from Merk Sigma) to inhibit the Complex I and III of the electron transport chain, respectively. Immediately upon the treatments, OCR dye and HS Mineral Oil were added to each well and the readings were made in dual-read TRF mode (Victor^3^V, Perkin Elmer Inc., Waltham, MA, USA) every 2 minutes for at least 45 minutes, according to manufacturer's instructions.

For ECAR measurements, moDCs were washed in the unbuffered DMEM (wo bicarbonate, with 2 mM L-glutamine and 143 mM NaCl) and plated as in the OCR assay. In addition to basal ECAR, each moDC culture was treated with 10 mM glucose to induce glycolysis, 2 μM oligomycin for maximal glycolysis induction, or 20 mM 2-DG for glycolysis inhibition. Immediately upon the treatments, ECAR dye was added, and the readings were made in dual-read TRF mode every 2 minutes for at least 45 minutes, according to the manufacturer's instructions. The lifetime fluorescence for OCR and ECAR measurements and the corresponding curve slopes were calculated in the provided Data Visualization Tool V 1.27 (Agilent Technologies) upon normalization of values to cell-free controls. The relative lifetimes were expressed as the ratio of each time-point measurement and the first measurement. Spare respiratory capacity (SRC) was calculated as the OCR ratio between maximal respiration in the presence of FCCP and basal respiration. Glycolytic capacity was calculated as the ECAR ratio between the maximal glycolysis (Oligomycin) and the basal glycolysis, whereas glycolysis rate was calculated as the ratio between ECAR after the glucose treatment and the basal glycolysis.

### Flow cytometry

Flow cytometry was used to assess the phenotype of moDCs and T cells after the cultures. The cells were washed once in PBS/ 2% FCS/ 0.01% Na-azide (Sigma-Aldrich) and incubated with Human TrueStain FcX (BioLegend) for 15 minutes prior to labelling them with fluorochrome-conjugated monoclonal antibodies (clones) at the dilutions recommended by the manufacturer. The full list of antibodies and the protocol is provided in Supplement Materials and Methods.

### Quantitative polymerase chain reaction

Total RNA was extracted from moDCs differentiated in the presence or absence of α-KG (50 mM) and matured or not with LPS/IFN-γ. The RNA was extracted using the Total RNA Purification Mini Spin Kit (Genaxxon Bioscience GmbH, Ulm, Germany) according to the manufacturer's instructions. The cDNA was prepared by reverse-transcription from 50 ng of total isolated RNA using a High-Capacity cDNA Reverse Transcription Kit (Thermo Fisher Scientific). The amplification of the synthesised cDNA was carried out in a 7500 real-time PCR system (Applied Biosystems, Massachusetts, United States) using the SYBR Green PCR Master Mix (Thermo Fisher Scientific). The amplification was carried out by denaturation at 95 ℃ for 10 min, followed by 40 cycles of 95 ℃ for 30 sec, 58 ℃ for 30 sec and 72 ℃ for 30 sec. PCR primers used are provided in Supplementary Materials and Methods. The results were normalised against the GAPDH gene and expressed as relative target abundance using the 2^-ΔΔCt^ method. All reactions were run in triplicates.

### Western blot

MoDCs were collected after 2 or 5 days of cultivation in the presence or absence of α-KG (50mM) and LPS/IFN-γ, as described 37. Protein concentration was measured with the BCA Assay Kit (Thermo Fisher Scientific) and 20 μg of extracted proteins were separated on 12% SDS-PAGE and transferred to a 0.2 mm nitrocellulose membrane (GE Healthcare). Western blotting was performed overnight at 4 °C with antibodies against anti-FoxO1 (C29H4) and anti-Akt1/2/3 (9272) (products of Cell Signalling Technology, USA), anti-FoxO1/FKHR (Ser256) (NB100-81927) (product of Novus Biologicals USA), anti-phospho-Akt1/ 2/3 (Ser473) (sc-7985-R) (product of Santa Cruz Biotechnology, Inc., USA) and anti-GAPDH (product of Invitrogen). Chemiluminescence was detected using the ChemiDoc Touch Imaging System with Image Lab Touch Software 6.1 (Bio-Rad, Hercules, CA, USA). The density of the bands was quantified in ImageJ 1.50f (National Institutes of Health, NIH, Bethesda, MD, USA) software. The results for pFoxO1, totalFoxO1, pAkt, and totalAkt in all samples were normalized to GAPDH, and all values were normalized to control moDCs collected after 2 days 1.

### αKG and cytokine measurements

The extracellular and intracellular levels of αKG were measured on day 2 and day 5 of moDCs cultures in cell-free culture supernatants and deproteinised cell lysates, by using the colourimetric and fluorometric alpha-KG assay kit, respectively, according to manufacturer's instructions (Abcam, Cambridge, UK). Prior to deproteinization, the total protein levels in cell lysates were determined with Pierce™ BCA Protein Assay Kit (Thermo Fisher) according to the manufacturer's instructions.

The supernatants from PHA-stimulated PBMC cultures were collected after 48h and assayed for cytokine measurements using the LEGENDplex™ Essential Immune Response panel (BioLegend). The supernatants from moDC cultures were assayed using LEGENDplex™ human Macrophage/Microglia panel (BioLegend), IL-27 and moDC/T cell co-cultures supernatants were analysed using specific sandwich enzyme-linked immunosorbent assay (ELISA) for IL-4, IFN-γ, IL-17, IL-10 and TGF-β (all from R&D Systems). All cytokine measurements were done in duplicates and the concentrations were calculated according to 5-parameter nonlinear fit curves (GraphPad Prism 8).

### Statistical Analysis

To analyse the differences between the αKG-treated and control groups, a repeated-measures one-way or two-way analysis of variance (RM-ANOVA) with Geisser-Greenhouse's correction for sphericity was performed, followed by Dunnett's or Tukey's multiple comparison test, respectively (GraphPad Prism 8). Data are presented as means ± SEM of the indicated number of independent experiments and a 95% confidence interval was taken as a significant difference between the tested groups. The heat map for cytokine concentrations was prepared by ranging the values from each experiment from 0-1 according to the formula: range value = (value - minimal value) / (maximal value - minimal value).

## Results

### Exogenous α-ketoglutarate displays immunomodulatory effects in PBMC cultures

To study the immunomodulatory properties of exogenous αKG, we first excluded the doses which are toxic in cultures with human PBMCs. αKG was applied at doses of 1.2 mM - 200 mM for 24h (not shown) and 48h Supplement Figure 1. MTT assay suggested that the doses of 100 mM and 200 mM significantly reduced MTT %, whereas the lower doses did not affect MTT % Supplement Figure 1A). The analysis of dead (predominantly necrotic) cells suggested that both 100 mM and 200 mM αKG induced necrosis. When the % of PBMCs with hypodiploid nuclei (late apoptotic cells) was analysed, the highest % of late apoptotic cells was detected in cultures with 100 mM αKG (Supplement Figure 1B-D). These results suggested that 200 mM induced predominantly necrosis, whereas 100 mM αKG induced both apoptosis and necrosis of PBMCs.

Since ROS are the key regulators of cell survival and death (38, we next analysed intracellular ROS content in living PBMCs treated with αKG for 48h, by using superoxide-anion and H_2_0_2_ indicator DHE Supplement Figure 1E-F). It was found that the doses of 100 mM and 200 mM αKG induced a significant increase in DHE fluorescence. The doses of 50 mM somewhat increased % of DHE^+^ PBMCs, but the differences were not significant compared to the control non-treated PBMCs. In contrast, the treatment of PBMCs with doses lower than 50 mM αKG did not affect intracellular ROS levels.

**Figure 1:**
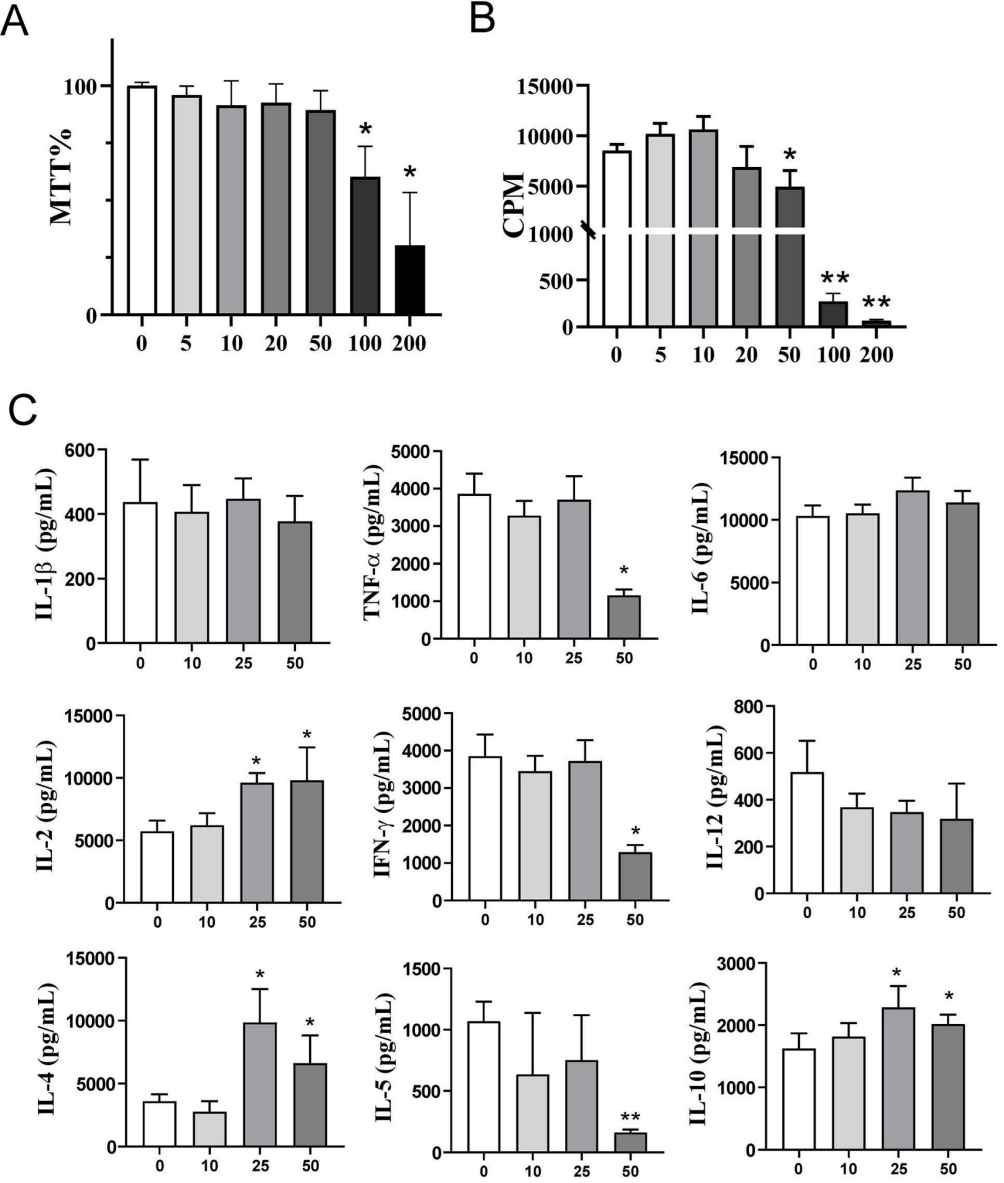
Effects of exogenous αKG on PHA-stimulated PBMC viability, proliferation, and cytokine production. PBMCs (3x10^5^ cells/well) were cultivated in the presence or absence of αKG (5 - 200 mM), and PHA (10 μg/ml) for 72h. **A)** The relative metabolic activity (MTT %) was determined in the MTT assay after the cultures, and the data is shown as mean MTT% ± SEM (n=3 different donors). **B)** The proliferation of PBMCs, according to [^3^H]-thymidine incorporation during the last 18h of culture, was determined by β-scintillation counting, and the data is shown as counts per minute (CPM) ± SEM (n=3 different donors). **C)** The cytokines produced in PHA-PBMCs cultures were determined from culture supernatants, and the data is shown as mean pg/ml ± SE of four independent experiments with different PBMC. **(A-C)** *p<0.05, **p<0.01 compared to control (ctrl) (RM-ANOVA, Dunnett post-test).

We next investigated how exogenous αKG affects the proliferation and cytokines production by PBMCs stimulated by polyclonal activator PHA. Thereby, the toxicity profile of αKG in PHA-PBMCs cultures after 72h according to % MTT (Figure 1A) was similar to that in PBMC cultures without PHA (Supplement Figure 1A). The proliferation of PHA-PBMCs in cultures treated with 200 mM, 100 mM and 50 mM αKG was reduced significantly compared to control PHA-PBMCs cultures, whereas the lower doses did not affect the proliferation of PHA-PBMCs (Figure 1B). We also measured the levels of cytokines in the supernatants of cultures treated with αKG (Figure 1C) and found that the cultures treated with 25 mM αKG contained more IL-2, IL-4 and IL-10 as compared to control PHA-PBMCs. The cultures treated with 50 mM αKG contained significantly less TNF-α, IFN-γ and IL-5, and higher levels of IL-2, IL-4 and IL-10, as compared to control PHA-PBMC cultures. These results suggested that exogenous αKG modulates the proliferation and cytokines production of polyclonally stimulated PBMCs at non-toxic doses.

### Exogenous α-ketoglutarate alters differentiation and maturation of monocyte-derived dendritic cells

**Figure 2:**
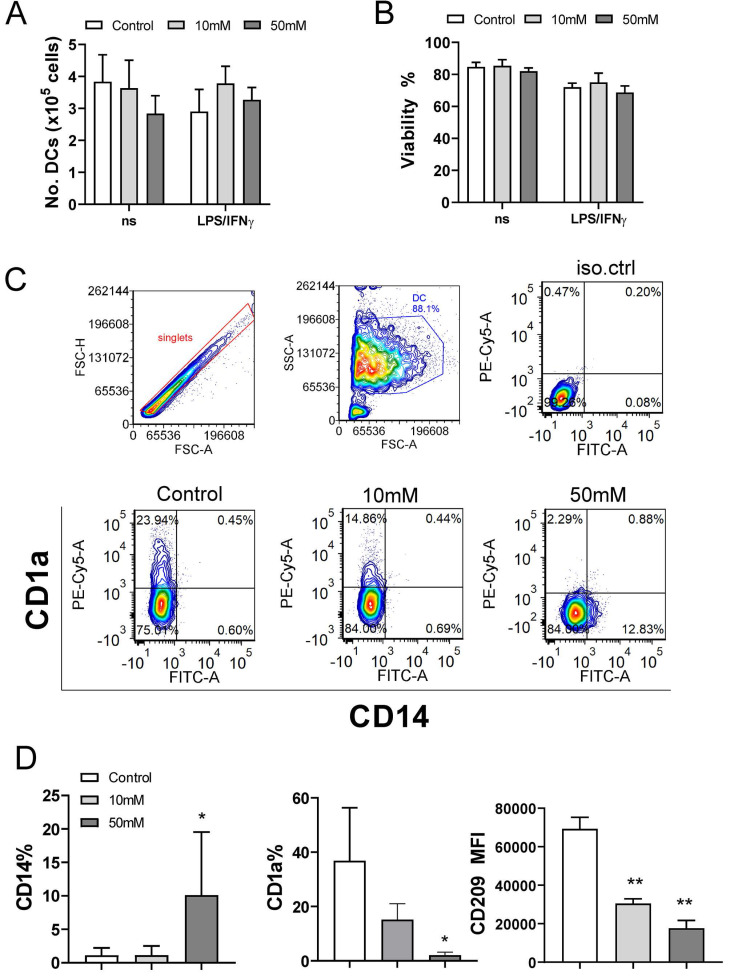
Effects of exogenous αKG on viability and differentiation of MoDCs. MACS-purified CD14^+^ monocytes (1x10^6^/ well of 6-wells plate) were cultivated in the presence of 20ng/ml GM-CSF and IL-4, and αKG (10 mM or 50 mM) or its absence for 4 days, followed by the stimulation with LPS/IFN-γ, or not, for the next 16-18h. **A)** The number of viable moDCs harvested after the cultures on day 5, and **B)** the percentage of their viability, were determined with Cell count and viability kit on Muse Cell Analyser, and the data are shown as mean ± SEM (n=4 different donors). **C)** A representative gating strategy and contour plots of CD1a/CD14 co-expression in gated DCs are shown, and **D)** the summarised data on the % of CD14^+^ moDCs, % of CD1a^+^ moDCs, and mean fluorescence intensity (MFI) of CD209 expression (>90% of gated cells expressed CD209) is shown as mean ± SEM (n=4 different donors). *p<0.05, **p<0.01 compared to control (RM-ANOVA, Dunnett post-test).

**Figure 3:**
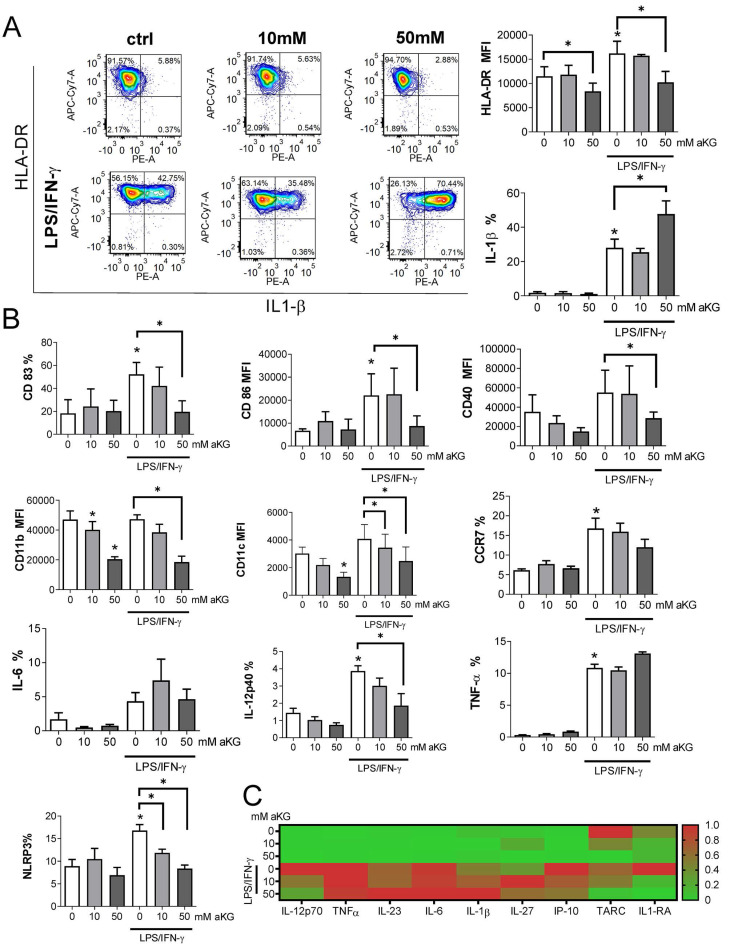
Maturation capacity of αKG-differentiated moDCs. MoDCs were generated with GM-CSF and IL-4, either in the presence of αKG (10 mM or 50 mM) or its absence for 4 days, followed by the stimulation with LPS/IFN-γ, or not, for the next 16-18h. **A)** A representative analysis of HLA-DR/IL-1β co-expression on immature (upper panel) or mature (lower panel) moDCs is shown as contour plots, along with the summarised data as mean (MFI or %) ± SEM, from 4 independent experiments with different donors. **B)** The expressions of CD83, CD86, CD40, CD11b, CD11c, CCR7, IL-6, IL-12p40, TNF-α and NLRP3, as determined from the same experiments by flow cytometry, are shown as mean (MFI or %) ± SEM (n=4). **C)** The cytokine levels, presented in a heat map with ranged values for each cytokine (0-1), were determined from moDCs culture supernatants on day 5 by flow cytometry or ELISA for IL-27 (the summarized data in pg/ml is presented in Supplement Figure 1). **(A, B)** *p<0.05 compared to control immature moDCs (0 mM αKG), or as indicated (RM-ANOVA, two-way, Tukey's post-test).

The proliferation and cytokines production in the PHA-PBMCs model largely depend on the costimulatory functions of APCs within PBMCs (39. To investigate the effects of exogenous αKG on APCs in more detail, we next used models of human moDCs generated in vitro, and their interactions with T cells*.* Thereby, immature moDCs were differentiated either in the presence of αKG (10 mM or 50 mM) or its absence, followed by stimulation with LPS/IFN-γ to induce mature moDCs. The total number and viability of immature and mature moDCs collected on day 5 were not affected significantly during the cultivation in the presence of αKG (Figure 2A, B). However, we observed significant alterations in the phenotype of moDCs differentiated with αKG. Namely, moDCs differentiated with 50 mM αKG displayed significantly reduced expression of CD1a and CD209, compared to control moDCs, as well as higher expression of CD14 (up to 20% of cells) (Figure 2C, D). Even when differentiated with the lower dose of αKG (10 mM), moDCs showed a reduced CD1a expression and significantly lower expression of CD209 compared to control immature moDCs. Further analysis showed that immature αKG-moDCs also had a lower expression of HLA-DR (Figure 3A), CD11b and CD11c (Figure 3B), whereas other markers were not affected significantly.

To evaluate the maturation capacity of αKG-differentiated and control moDCs, the cells were stimulated with LPS/IFN-γ which are known to induce their Th1 polarising capacity 19,24,40. Expectedly, LPS/IFN-γ up-regulated the expression of HLA-DR, IL-1β, CD83, CD86, CD40, CCR7, IL-12, NLRP3 and TNF-α by control moDCs. However, moDCs differentiated in the presence of 50mM αKG displayed significantly lower potential to up-regulate HLA-DR, CD83, CD86, CD40, NLRP3 and IL-12 expression compared to control moDCs (Figure 3A, B).

Interestingly, mature αKG-moDCs displayed an unaltered capacity to express IL-6 and TNF-α, and a significantly higher capacity to express IL1-β compared to control mature moDCs (Figure 3A). A similar phenomenon was observed when the levels of cytokines were measured in cell culture supernatants, wherein significantly lower levels of IL-12p70 were detected, unaltered levels of TNF-α, IL-6 and IL-23, and increased levels of IL-1β (Figure 3C, Supplement Figure 2). Besides, mature αKG-moDCs displayed a lower capacity to secrete IP-10 (CXCL10), as well as IL-1RA Supplement Figure 2 compared to control mature moDCs. These results suggest that the impaired differentiation of moDCs in the presence of αKG severely affected their subsequent maturation potential, but the capacity of αKG-moDCs to produce innate pro-inflammatory cytokines upon maturation was maintained and probably independent of NLRP3 expression.

### Exogenous α-ketoglutarate up-regulates anti-oxidative protection and increases autophagy flux in moDCs

**Figure 4:**
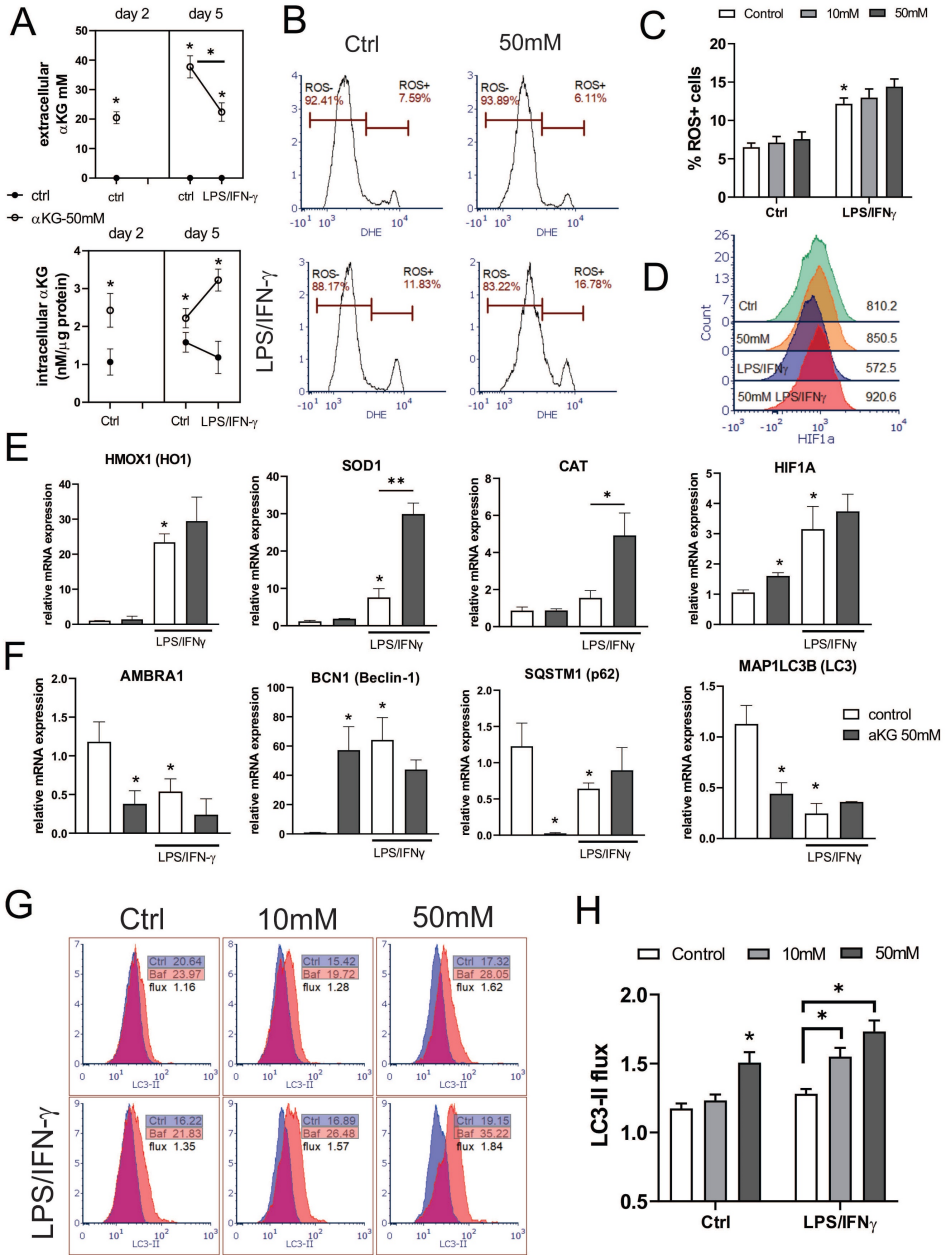
Regulation of ROS and autophagy in αKG-differentiated moDCs. MoDCs were generated with GM-CSF and IL-4, either in the presence of αKG (10 mM or 50 mM) or its absence for 4 days, followed by the stimulation with LPS/IFN-γ, or not, for the next 16-18h. **A)** αKG was measured in cell-free culture supernatants (extracellular αKG) and in deproteinised cell lysates (intracellular αKG), by colourimetric and fluorometric assay kit, respectively. The intracellular concentrations of were normalised to the total protein levels. The data is shown as mean ± SD of 2 independent experiments. **B)** Representative histograms are shown, of intracellular ROS measured in moDCs by DHE staining, and **C)** the summarised data on % of ROS+ cells are shown as mean ± SEM (n=3 different donors).** D)** The expression of HIF-1α protein levels was measured by flow cytometry, and the representative histograms from one experiment, out of three similar results indicate MFI of HIF-1α expressions in gated moDCs (the summarized data from all experiments is shown in Supplement Figure 2). **E)** The relative mRNA expression of genes involved in redox regulation, as measured by qPCR in moDCs is shown as mean ± SEM (n=3). **F)** The relative mRNA expression of genes involved in autophagy regulation is shown as mean ± SEM (n=3). **G)** Representative histograms on autophagy flux measurements, representing the ratio of membranous LC3-II expression in Bafilomycin-treated (Baf) and non-treated (ctrl) moDCs, are shown along with indicated MFIs. **H)** The summarised data on autophagy flux (LC3-II flux) is shown as mean ± SEM (n=3). **(B, C, G, E)** *p<0.05 vs control immature moDCs, or as indicated (RM-ANOVA, two-way, Tukey's post-test).

As a regulator of cellular metabolism, αKG is also involved in the regulation of intracellular ROS 41, which are detrimental to moDCs differentiation and functions 28. Therefore, we measured extracellular and intracellular concentrations of αKG in moDCs cultures treated with 50 mM αKG on day 2 and day 5 (Figure 4A). Expectedly, higher intracellular levels of αKG were found in αKG-moDCs compared to control moDCs on both day 2 and day 5. Interestingly, significantly higher intracellular αKG levels were observed in αKG-moDCs treated with LPS/IFN-γ compared to immature αKG-moDCs. This increase was followed by a reduction in exogenous αKG levels, as measured in the supernatants of αKG-moDCs cultures, suggesting an uptake of extracellular αKG during the moDCs maturation.

Several enzymes in redox regulation were demonstrated to be involved in the regulation of DC maturation and functions 25-27. Therefore, we analysed how αKG modulates oxidative status in immature and LPS/IFN-γ-matured moDCs (Figure 4). When the intracellular ROS levels were measured during moDCs differentiation on day 2, αKG-treated moDCs displayed an increased level of ROS, compared to control moDCs Supplement Figure 3A, B). However, on day 5, immature αKG-treated and control moDCs showed no significant differences in ROS (Figure 4B, C). The protein expression of HIF-1α (Figure 4D), as well as the levels of mRNA for HO-1, CAT and SOD, were unaffected by αKG in immature moDCs on day 5. On the other hand, HIF-1α mRNA was upregulated in immature αKG-moDCs compared to control moDCs. The stimulation of moDCs with LPS/IFN-γ increased the levels of intracellular ROS, and mRNAs for HO-1, HIF-1α and SOD, whereas it decreased HIF-1α protein levels in control moDCs. However, αKG-moDCs displayed a drastically higher expression of mRNA for CAT and SOD after the LPS/IFN-γ stimulation compared to control moDCs, as well as higher HIF-1α protein levels (Figure 4E, Supplement Figure 3. These results suggested that αKG prevented LPS/IFN-γ-induced degradation of HIF-1α during the maturation. In line with this, when the non-toxic doses of ROS-inhibitor NAC were present during the cultivation of moDCs, the levels of HIF-1α were somewhat reduced, and were similar between αKG-treated and control mature moDCs Supplement Figure 3B-D), which suggested that αKG did not directly prevent LPS/IFN-γ-induced degradation of HIF-1α.

Considering that the redox status and autophagy are mutually tightly regulated in DCs (28,42, we next measured the expression of genes involved in the regulation of autophagy (Figure 4F). The expression of the membranous fraction of LC3 (LC3-II) was measured in Bafilomycin-treated or non-treated cells to determine the autophagy flux in moDCs (Figure 4G, H). Immature αKG-moDCs displayed substantially lower levels of mRNA for LC3, p62/SQSTM1 and AMBRA1, and higher levels of Beclin 1 (BECN1) mRNA, compared to control immature moDCs (Figure 4F). According to lower mRNA for LC3, the expression of membranous LC3-II protein was also reduced in αKG-moDCs. However, we observed a higher LC3-II flux in αKG-moDCs, when compared to the flux in control immature moDCs (Figure 4G, H). The stimulation of control moDCs with LPS/IFN-γ, lowered the mRNA expression for p62/SQSTM1, AMBRA1, LC3, and LC3-II protein expression, whereas it increased BECN1 mRNA expression. In contrast, p62/ SQSTM1 mRNA was increased in αKG-moDCs upon the stimulation with LPS/IFN-γ, without significant changes in mRNA for LC3, AMBRA1 and BECN1 (Figure 4F). Thereby, mature αKG-moDCs also displayed a significantly higher LC3-II flux compared to control mature moDCs. This phenomenon was directly proportional to the dose of αKG used in moDCs cultures and was not affected by LPS/IFN-γ stimulation. Moreover, the treatment of moDCs with ROS inhibitor during their differentiation, inhibited the capacity of αKG to increase the autophagy flux in moDCs Supplement Figure 3E), suggesting that this effect of αKG was related to αKG-induced changes in the oxidative metabolism.

Both redox potential and autophagy are critically regulated by the FoxO1 transcription factor and its upstream negative regulator protein kinase B (Akt) (43,44. Therefore, we measured the expression of phosphorylated (p)Akt-Ser 473 and total Akt, as well as pFoxO1-Ser 256 and total FoxO1 by Western Blot in moDCs from 2-day or 5-day cultures Supplement Figure 4. After two days of cultivation, αKG-treated moDCs showed higher expressions of Akt, pAkt, FoxO1 and pFoxO1 compared to control moDCs. After 5 days of cultivation, immature αKG-moDCs displayed somewhat reduced pAkt and total Akt expression compared to control moDCs, whereas the pAkt/ total Akt ratio was similar between the two groups, even after their stimulation with LPS/IFN-γ. In contrast, the total expression of FoxO1 decreased, and an increased ratio of pFoxO1/ total FoxO1 was observed in αKG-moDCs compared to control moDCs, especially after their stimulation with LPS/IFN-γ. These results indicated the reduction of FoxO1 transcriptional activity in αKG-moDCs.

### Exogenous α-ketoglutarate increases OXPHOS and glycolysis in moDCs

**Figure 5:**
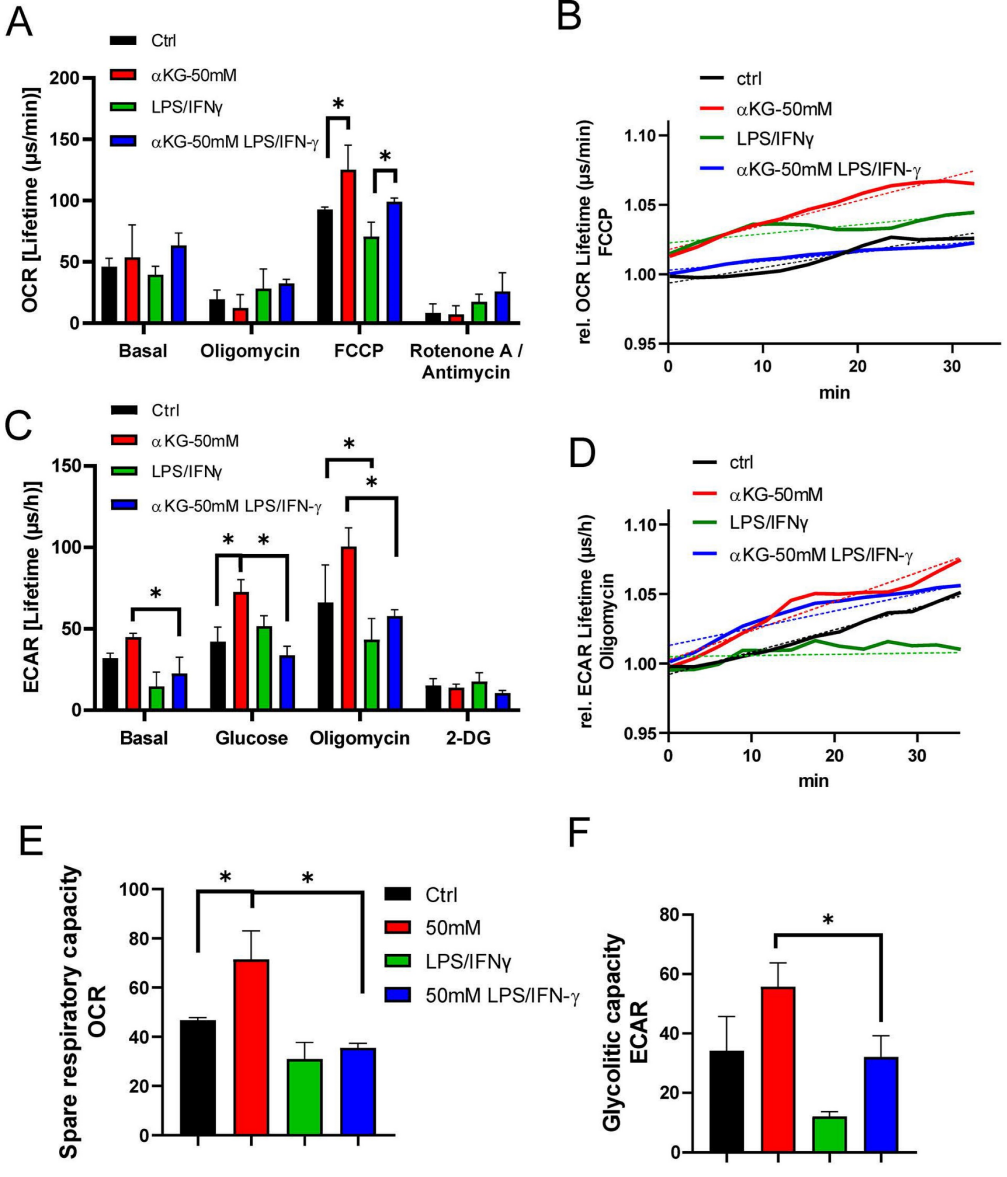
Oxygen consumption rate (OCR) and extracellular acidification rate (ECAR) in αKG-moDCs. MoDCs were generated in the presence of αKG (50 mM) or its absence, and stimulated with LPS/IFN-γ or not. **A)** OCR, expressed as lifetime μs/min, was measured every 2 minutes in moDCs (5x10^5^ cells/well) in the basal conditions, or in the presence of FCCP (0.5 μM), oligomycin (2 μM) or Rotenone (1 μM) /Antimycin (1 μM), simultaneously for at least 45 minutes, and was calculated from the lifetime curve slopes. **B)** The representative curves of relative OCR in the presence of FCCP (full lines) with the non-linear fit curves (dotted lines) are shown. **C)** ECAR, expressed as lifetime μs/h, was measured in moDCs (5x10^5^ cells/well) in the basal conditions, or in the presence of additional glucose (10 mM), oligomycin (2 μM) or 2-DG (20 mM) simultaneously, and calculated from the lifetime curve slopes. **D)** The representative curves of relative ECAR in the presence of oligomycin (full lines) with the non-linear fit curves (dotted lines) are shown. **(A, C)** The data is shown as mean ± SD of triplicates from one experiment, out of three with similar results. **E)** SRC and **F)** Glycolytic capacity, were calculated as the ratios between maximal and basal OCR and ECAR, respectively, and shown as mean ± SEM from 3 independent experiments. (A, C, E, F) * p<0.05 as indicated (RM-ANOVA, two ways, Tukey's post-test).

Both autophagy and oxidative status are critically regulated by mitochondria respiration 45, and previous data suggested that αKG-derivates could directly affect OXPHOS and glycolysis in bone-marrow-derived macrophages 31, as well as that Akt/FoxO1 signalling was critically involved in these processes 46. Therefore, to better understand how αKG modulated oxidative metabolism, we measured OCR and ECAR in moDCs after the differentiation and maturation, as well as SRC, glycolytic rate and glycolytic capacity (Figure 5). Real-time monitoring of OCR suggested that the basal respiration in αKG-moDCs was slightly, but not significantly, increased compared to control moDCs. This phenomenon was more obvious in the presence of FCCP which induces maximal respiration. Generally, immature moDCs displayed a higher OCR than the corresponding mature moDCs (Figure 5A, B).

The mitochondrial complex V inhibition with oligomycin, as well as the blockage of the electron transportation chain with Rot/AA, reduced OCR in all cultures. SRC, calculated as the ratio between maximal and basal OCR, was the highest in immature αKG-moDCs, and it was significantly down-regulated after their maturation with LPS/IFN-γ (Figure 5D). To observe how the inhibition of OXPHOS affected redox regulation and autophagy in moDCs, non-toxic doses of Rot/AA were added to moDCs cultures on day 2 Supplement Figure 3B), and the levels of ROS, HIF-1α and autophagy flux were monitored on day 5. In the presence of Rot/AA, control immature moDCs increased the intracellular levels of ROS (Supplement Figure 3B) unlike αKG-moDCs. Additionally, the increase of autophagy flux and the stabilisation of HIF-α upon LPS/IFN-γ stimulation in αKG-moDCs was impaired in the presence of Rot/AA (Supplement Figure 3F, H). However, the inhibition of OXPHOS by Rot/AA significantly reduced the levels of HIF-1α in both control- and αKG-moDCs (Supplement Figure 3F). Moreover, Rot/AA significantly modulated the expression of differentiation (CD1a, CD14 and CD11c) and maturation markers on moDCs (CD86, CD40, HLA-DR, PD1L, CCR7, secretion of IL-1β, IL-23, IL-12p70 and IL-10) (data not shown), making it unclear which of these αKG effects depend exclusively on OXPHOS.

Real-time monitoring of ECAR suggested that immature moDCs had higher basal glycolysis than the corresponding mature moDCs, and the differences were significant for αKG-moDCs (Figure 5C, D). More obvious differences were observed at the maximal glycolysis induced by oligomycin. After the glycolysis induction with 10 mM glucose treatment, all cells increased the glycolysis rate, expressed as the ratio between glucose-induced and basal glycolysis. The highest increase in glycolysis rate was detected in control mature moDCs (⁓3.5 times), followed by immature αKG-moDCs (⁓1.6 times), mature αKG-moDCs (⁓1.5 times) and immature moDCs (⁓1.3 times). Expectedly, 2-DG inhibited glycolysis in all moDCs cultures. The analysis of glycolytic capacity, as the ratio of maximal and basal ECAR, revealed that immature αKG-moDCs had the highest glycolytic capacity, followed by immature control moDCs, mature αKG-moDCs and mature control moDCs, respectively (Figure 5F).

### α-ketoglutarate-moDCs display reduced Th1, but increased Th2 and Th17 polarizing capacity after the maturation

**Figure 6:**
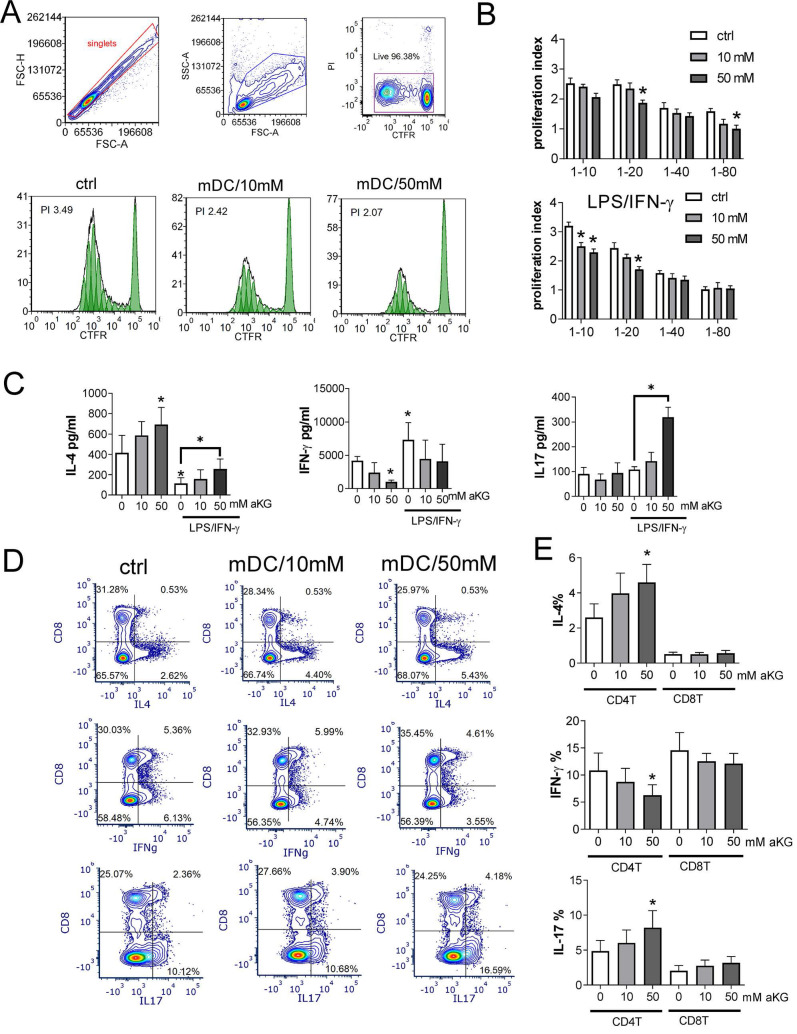
Allostimulatory and T cell polarising capacity of αKG-moDCs. MoDCs (1x10^4^-0.12x10^4^ cells/well) were co-cultured with CFTR-labelled allogeneic T cells (1x10^5^) (1:10-1:80 moDC: T cell ratio, respectively) for 4 days. **A)** The proliferation of T cells, according to CFTR dilution, was monitored by flow cytometry upon the exclusion of doublets and dead (PI+) cells, as indicated in representative histograms displaying T cell proliferation in co-cultures with LPS/IFN-γ-stimulated moDCs (mDCs) (1:10 mDC: T cell ratio). **B)** The summarised data on T cell proliferation in the presence of immature or LPS/IFN-γ-matured moDCs is shown as mean proliferation index ± SEM (n=3). **C)** The levels of IL-4, IFN-γ and IL-17, detected in moDC: T cell co-culture supernatants (1:20 moDC: T cell ratio) were determined by ELISA, and the data is shown as mean pg/ml ± SEM (n=3 pairs of unrelated moDCs and T cell donors). **D)** A representative analysis of intracellular cytokines is shown, within CD8^+^ and CD4^+^ (CD8^-^) T cells co-cultured with mature moDCs (mDCs) as in (C) and stimulated with PMA/ Ca ionophore/ monensin for the last 4h of cultures. **E)** The summarised data on % of cytokine expressing CD4+ (CD8-) T cells, and CD8+ T cells are shown as mean ± SEM (n=3). **(B, C, E)** *p < 0.05 vs. corresponding control moDCs, or as indicated (RM ANOVA, two-way, Tukey's post-test).

Considering the observed effects of αKG on oxidative and glycolytic metabolism, we next wondered how this is reflected in the capacity of moDCs to induce allogeneic proliferation of T cells and their polarisation into effector subsets. Thereby, the allogeneic co-culture model was used to abstract the complex interactions between DCs and different antigens (47 and to increase the percentage of T cells reactive to allogeneic MHC molecules *in vitro* 48. αKG-moDCs, particularly those treated with 50 mM αKG, displayed a reduced allostimulatory capacity compared to control moDCs, which was more pronounced after their stimulation with LPS/IFN-γ (Figure 6A, B).

The measurements of IL-4, IFN-γ and IL-17 levels in moDCs/T cell co-culture supernatants, and their normalisation to the same number of T cells in co-cultures, suggested that the co-cultures with immature moDCs contained higher levels of IL-4, and lower levels of IFN-γ than the co-cultures with mature moDCs (Figure 6C). Thereby, the co-cultures with αKG-moDCs repeatedly contained higher levels of IL-4, and lower levels of IFN-γ, than the co-cultures with control moDCs. Interestingly, co-cultures with mature αKG-moDCs displayed significantly higher levels of IL-17 than the corresponding control moDCs.

To find which T cell subset was predominantly responsible for the observed changes in cytokines production, we analysed intracellular cytokines within CD4^+^ and CD8^+^ T cell subsets (Figure 6D). The analysis revealed that the differences in T polarising capacity between moDCs and αKG-moDCs were predominantly related to their effects on CD4^+^T cells, rather than CD8^+^T cells. Namely, we found a significantly higher % of IL-4^+^ and IL17^+^ CD4^+^T cells, and a lower % of IFN-γ^+^CD4^+^T cells in co-cultures with mature αKG-moDCs compared to control mature moDCs (Figure 6D). A similar phenomenon was observed when transcription factors GATA3, T-bet and ROR-γt were analysed in CD4^+^ T cells, as well as when MACS-purified CD4^+^T cells were used in the co-cultures instead of total allogeneic T cells, although somewhat lower % of cytokine-producing cells was observed Supplement Figure 5. Thereby, we did not observe a significant contribution of Th cells that co-express IL-4/IL-17, IL-17/IFN-γ or IL-4/IFN-γ to the differences between control and αKG-moDCs. These results suggested that αKG-moDCs display reduced Th1- and increased Th2-polarizing capacity, irrespective of their maturation status, while they gain additional Th17-polarization capacity after the maturation with LPS/IFN-γ.

### Immature α-ketoglutarate-moDCs induce conventional regulatory T cells via IDO-1

**Figure 7:**
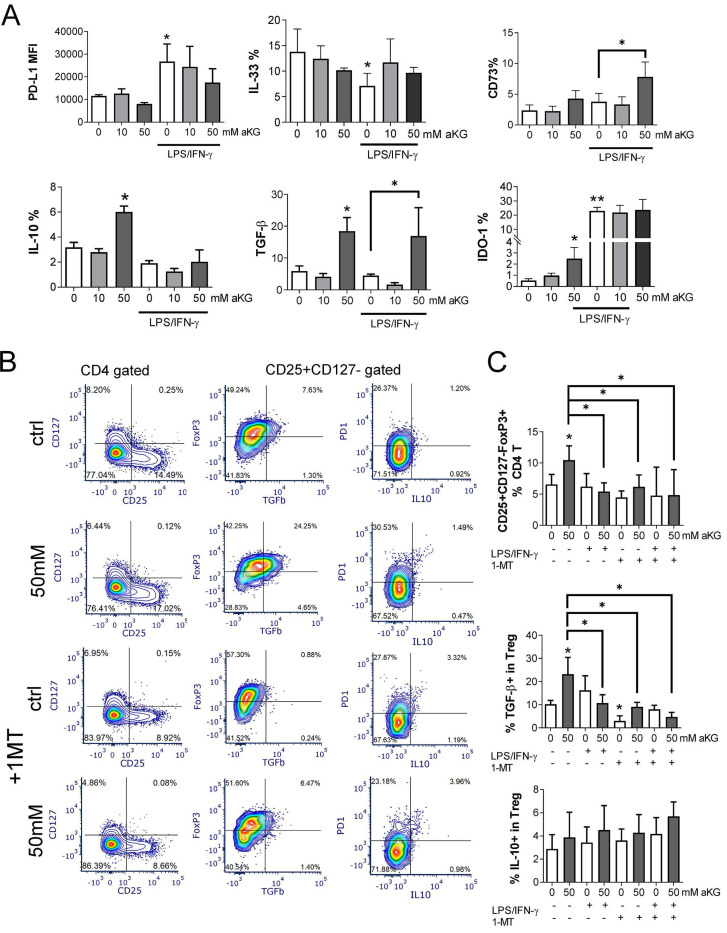
The tolerogenic potential of mature and immature αKG-moDCs. MoDCs were generated with GM-CSF and IL-4, either in the presence of αKG (10 mM or 50 mM) or its absence for 4 days and stimulated with LPS/IFN-γ or not. **A)** The expression of tolerogenic surface (PD-L1, CD73) and intracellular (IL-33, IL-10, TGF-β, IDO-1) markers by moDCs were analysed on day 5 by flow cytometry and the data are shown as mean (MFI or % of positive cells) ± SEM of n=4 independent experiments each carried out with moDCs from different donors. **B)** A representative analysis of conventional Tregs induced in 6-day moDCs/CD4^+^ T cell co-cultures in the presence of immature αKG-moDCs or control moDCs and IL-2, and either in the presence of IDO-1 blockade (1MT) or its absence, is shown. After dead cells and doublets exclusion (not shown), the CD25+CD127- cells were gated within CD4+T cells, and within these cells, FoxP3/TGFβ or PD1/IL-10 co-expressions were analysed.** C)** The summarised data from three independent experiments, each carried out with different moDC/T cell pairs, on the total % of FoxP3^+^CD25^+^CD127^-^ cells (Tregs) within CD4+ T cells is shown as mean ± SEM, along with the % of TGF-β^+^ cells or IL-10^+^ cells within the Tregs. **(A, C)** *p<0.05, **p<0.01 vs. control immature moDCs, or as indicated (RM ANOVA, two-way, Tukey's post-test).

Increased OXPHOS, glycolysis, autophagy and Th2-shifted polarization all point to tolerogenic properties of DCs 17,19,20,23. Considering the results of this study, we further evaluated the tolerogenic potential of αKG-moDCs by analysing the expression of key tolerogenic markers on immature and LPS/IFN-γ-matured moDCs (Figure 7A). Immature moDCs displayed lower expression of PD-L1 and IDO-1, and a higher expression of IL-33, compared to mature moDCs, whereas CD73, TGF-β and IL-10 were similarly expressed on immature and mature moDCs. Thereby, immature αKG-moDCs, especially those differentiated with 50 mM αKG, displayed significantly increased expression of IL-10, TGF-β and IDO-1, compared to control immature moDCs. However, after the maturation with LPS/IFN-γ, these cells showed only increased expression of TGF-β and CD73, whereas other markers in this panel were not different from control mature moDCs (Figure 7A).

We also co-cultivated allogeneic CD4^+^T cells and moDCs at suboptimal ratio (moDCs: T cell ratio at 1:50) in the presence of low IL-2 dose to favour the differentiation of conventional Tregs 49, and identified them as CD4^+^CD25^+^CD127^-^Foxp3^+^ T cells according to previous data 50. The results showed that immature αKG-moDCs induced a higher proportion of conventional Tregs compared to control immature moDCs. Additionally, a higher proportion of αKG-moDCs- Tregs expressed TGF-β, but not IL-10 (Figure 7C) or PD1 (Figure 7B), compared to Tregs induced by control immature moDCs. Moreover, when we blocked the activity of IDO-1 with 1-MT in co-cultures, immature αKG-moDCs lost the capacity to induce a higher proportion of TGF-β^+^ Tregs. However, the capacity of αKG-moDCs to induce conventional Tregs and their TGF-β expression was lost after the stimulation of moDCs with LPS/IFN-γ, irrespective of 1-MT (Figure 7B, C). These results suggested that immature αKG-moDCs induce a higher proportion of conventional Tregs via IDO-1 and that this property is diminished upon their maturation with LPS/IFN-γ.

### α-ketoglutarate-moDCs induce IL-10-producing CD8 and Tr1 cells via ILT-3

**Figure 8:**
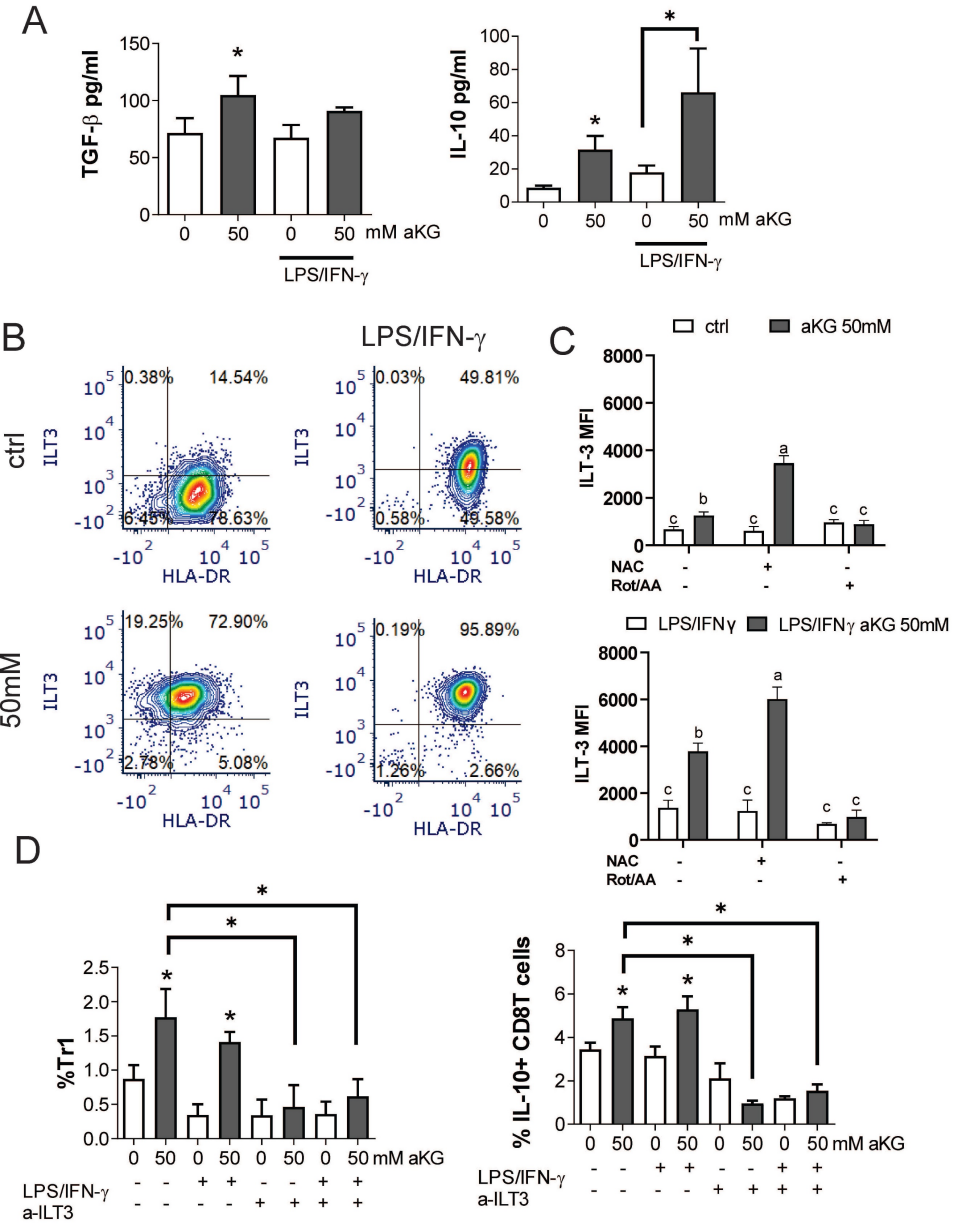
αKG-moDCs induce Tr1 and suppressor CD8+T cells via ILT3. **A)** The cytokines were analysed in moDCs/ T cell co-cultures carried out at 1:50 (moDC: T cell ratio) in the presence of IL-2 for 6 days. The data is shown as mean pg/ml ± SEM from 3 moDC/T cell co-cultures carried out with different donors. **B)** A representative analysis of ILT3/HLA-DR co-expression within the gated moDCs harvested after the differentiation and maturation with LPS/IFN-γ, is shown. **C)** During the generation of moDCs in the presence of αKG (50 mM) or its absence, some cultures were carried out in the presence of NAC (10mM) or Rot (0.5μM)/ AA(0.5μM) during the last 3 days. The cultures were then stimulated with LPS/IFN-γ or not, for the next 16-18h. The summarized data on ILT3 expression is shown as mean MFI ± SEM (n=3 different moDC donors). **D)** The % of Tr-1 cells within CD4^+^ T cells, and IL-10^+^ CD8^+^ T cells within CD8^+^ T cells were analysed after the co-cultures of total T cells with moDCs as in (A), either in the presence of blocking anti-ILT3 (2 µg/ml) or isotype control Ab. The data is shown as mean % ± SEM (n=3 different moDC/T cell co-cultures). The gating strategies for Tr1 and IL-10^+^CD8^+^ T cells are shown in Supplement Figure 4. **(A, D)** *p<0.05, vs. control immature moDCs or as indicated, (RM ANOVA, Dunnett's post-test). **C)** Significance between the samples is labelled with compact display letters according to Tukey's post-test.

In addition to conventional Tregs, tolerogenic DCs can induce additional types of regulatory T cells, including the non-conventional IL-10-producing Tr-1 and regulatory CD8^+^T cells 51-54. Accordingly, when we measured the levels of TGF-β and IL-10 in the supernatants of co-cultures of CD4^+^ T cells with immature αKG-moDCs, both cytokines were increased, whereas only IL-10 was increased in co-cultures with CD4^+^ T cells and mature αKG-moDCs, as compared to corresponding control moDCs (Figure 8A). These results suggested that, although mature αKG-moDCs do not induce conventional Tregs, they might induce non-conventional Tregs. To test this, we analysed the expression of ILT-3 on moDCs, which was found highly up-regulated on αKG-moDCs compared to control moDCs (Figure 8B, C). The stimulation of these cells with LPS/IFN-γ further increased ILT-3 expression on both αKG-moDCs and control moDCs, but the increase was higher on αKG-moDCs. The up-regulation of ILT-3 on αKG-moDCs was not inhibited, but rather more pronounced, when the cells were differentiated in the presence of NAC, suggesting that ROS act inhibitory on αKG-mediated up-regulation of ILT-3. In contrast, the inhibition of OXPHOS by Rot/AA completely impaired the up-regulation of ILT-3 by αKG, irrespective of their maturation status (Figure 8D).

IL-10-producing Tr1 and suppressor CD8^+^ T cells were analysed from the co-cultures, as IFN-γ^-^IL-4^-^FoxP3^-^IL-10^+^ cells within the CD4^+^ and CD8^+^ T cells, respectively Supplement Figure 6. The results showed that αKG-moDCs indeed induced a higher proportion of both Tr1 and suppressor CD8^+^T cells compared to control moDCs. Furthermore, the stimulation with LPS/IFN-γ did not diminish this capacity of αKG-moDCs (Figure 8D). When moDCs/T cell co-cultures were carried out in the presence of blocking anti-ILT-3 Ab, the effect of αKG-moDCs on the induction of non-conventional CD4 and CD8 Tregs was diminished. These results suggested that αKG-moDCs were superior in the induction of Tr-1 and suppressor CD8^+^T cells via ILT-3 compared to control moDCs, and the phenomenon was stable throughout the maturation of moDCs.

## Discussion

Exogenous αKG was shown previously to display important physiological and pathophysiological roles 3,12,55,56, as well as a tremendous potential in supplementation immunotherapy 57. However, the mechanisms underlying its immunological effects remained poorly investigated, especially considering APC/T cell interactions which are vital for a successful immunotherapy. To the best of our knowledge, the models of PHA-stimulated human PBMCs and moDCs/T cell interactions were used here for the first time to assess αKG immunological mechanisms. To exclude the possibility of cytotoxicity in the observed immunomodulatory effects, we carefully selected non-toxic doses of αKG. A similar range of αKG doses was tested *in vitro* as in previous studies 8,58,59.

Zurek et al. 59 showed in LDH assay that αKG decreased the viability of human and mouse osteoblast cell lines, hFOB1.19 and MC3T3-E1, in doses ≥75 mM and ≥50 mM, respectively. Similar claims were made previously for the HT-29 cell line, where the doses of 50 mM and less were not cytotoxic 58. In line with these are our results showing that αKG was not cytotoxic for PBMCs and moDCs at < 50 mM, whereas 100 mM and 200 mM induced apoptosis and necrosis. The latter phenomenon is most probably mediated by ROS. Namely, Wu et al. 60 demonstrated that αKG induces autophagy and apoptosis upon induction of ROS and inhibition of mTOR in renal cell carcinoma cell lines, 786O and ACHN, in a dose-dependent manner. Moreover, the levels of ROS induced by different doses of exogenous αKG were involved in either prolonging (moderate induction of ROS) or shortening (high induction of ROS) the lifespans of different model organisms 61. The dose-dependent effects of ROS on apoptosis/necrosis turning point 62, could also explain why 200 mM αKG induced predominantly necrosis of PBMCs, whereas 100 mM induced both apoptosis and necrosis. It should be noted that the esterified analogues of αKG display even higher cytotoxicity *in vitro*, as demonstrated for HSC-T6 and BRL-3A cell lines, where doses ≥18 mM and ≥20 mM, respectively were cytotoxic 63. These data suggested that αKG display dose-dependent, cell-type-dependent, as well as analogue-dependent biological effects. Previous studies claimed that the esterified αKG analogues display a higher cell permeability 32,56,64. However, this view was challenged by the results of Parker et al. 35, who demonstrated that the esterified αKG analogues rapidly hydrolyse in aqueous media, yielding free αKG which is subsequently actively imported into many cell types and induce analogue-dependent, αKG-independent effects on cellular metabolism and functions 35. Accordingly, we also confirmed that the non-esterified form of αKG enters moDCs and increases its intracellular levels, especially after LPS/IFN-γ stimulation. Therefore, to exclude analogue-dependent effects of αKG, we used only the non-esterified form of αKG for the investigations.

αKG (50 mM) down-regulated IFN-γ, IL-5 and TNF-α production by PHA-PBMCs, and potentiated the production of IL-2, IL-4 and IL-10, whereas lower doses (25 mM) only upregulated the latter group of cytokines. The levels of IL-2 in supernatant are determined by its production and consumption by proliferating T cells 65. Therefore, the increased levels of IL-2 are probably a consequence of its lower consumption due to inhibited proliferation of PBMCs, as detected in the cultures treated with 50 mM αKG. This is also in line with the increased levels of IL-10, which can inhibit the proliferation of T cells directly 66 or indirectly via APCs 67. Moreover, IL-10 is known to inhibit pro-inflammatory and Th1 cytokines 68, which we observed as well. Interestingly, although IL-4 and IL-5 are both considered type 2 cytokines, their regulation and role in immune response are quite different 69. IL-4, together with IL-10, is considered a regulatory cytokine, and it is mainly involved in the induction of humoral response and the suppression of IFN-γ 57, 58. In contrast, IL-5 is involved in the activation of eosinophils and a proinflammatory response in asthma 72. Therefore, the effects of αKG on cytokine response by PHA-stimulated PBMCs could be interpreted as anti-inflammatory and immunoregulatory. Accordingly, Wu et al. 30 showed that the dietary uptake of non-esterified αKG down-regulated intestinal expression of proinflammatory markers TLR4, NF-kB and mRNA for TNF-α in *Cyprinus carpio* infected with *Aeromonas hydrophila*. A similar phenomenon was reported for glutaminolysis-derived αKG and dimethyl-αKG analogue in models of mouse bone-marrow-derived macrophages *in vitro* 31, and alveolar macrophages *in vivo*, in a model of acute respiratory distress syndrome 32. These papers suggested that αKG display anti-inflammatory properties and induce M2 polarization of macrophages via epigenetic and metabolic reprogramming of the cells. On the other hand, the findings of Klyzs et al. 33 suggested that the dimethyl-αKG analogue potentiates *T-bet* expression in T cells, favouriting their differentiation towards Th1 over Tregs. Therefore, it remained unclear how the observed effects in αKG-treated macrophages link with the subsequent T-cell response, especially considering the analogue-independent effects of αKG.

Here we described that exogenous αKG impairs the differentiation of human moDCs and their subsequent maturation. CD209 73, β2-integrin receptors CD11b and CD11c 74, HLA-DR and CD1a were all described as important differentiation markers regulating various aspects of DC functions 75. Thereby, CD1a was shown as a valuable marker that can predict for DC immunogenicity and their capacity to produce high levels of IL-12 76. In line with this, a weaker capacity of αKG-moDCs to up-regulate CD83, CD86, CD40, HLA-DR, CCR7, NLRP3 and IL-12 upon LPS/IFN-γ stimulation, was probably a consequence of their altered differentiation. A similar phenomenon was demonstrated previously, in cases of differentiation of moDCs for shorter periods of time 77, differentiation in the presence of tolerogenic nanomaterials 19,20, metabolism modulators such as VitD3 21, 2-DG 22 or Rotenone 29. Mangal et al. 34 reported that αKG-releasing microparticles (MP) increased the proportion of MHC class II^+^ CD86^+^ in mouse bone marrow-derived CD11c^+^ cells (from ~8% to ~12%) after the LPS treatment. In contrast, soluble αKG (0.1 μg/ml) reduced MHC class II^+^ CD86^+^ expression in CD11c cells (from ~8% to ~5%) 34, the latter of which resembles the phenomenon we observed. However, we demonstrated that the anti-inflammatory effects of exogenous αKG extend to an array of moDCs maturation and activation markers and their cytokines expression. Unlike IL-12, the capacity of αKG-moDCs to up-regulate innate cytokines (TNF-α, IL-6, IL-1β) upon stimulation was not impaired, whereas IL-1β expression was even increased. IL-1β is known to upregulate IL-6 and TNF-α production via IL-1R and MyD88/IRAK/TRAF6 pathway activating NF-kB 78, suggesting that these cytokines are mutually tightly regulated. Our results contrast the findings on suppressed expression of TNF-α, IL-6, IL-1β and IL-12 by mouse bone marrow-derived macrophages treated with dimethyl-αKG 31,32. The observed differences could be a consequence of different αKG analogues used these studies, but also a result of different regulation of cytokines in DCs and macrophages. Namely, the IL-12 productions by DCs and macrophages are differently regulated 79,80. Also, the activation of NLRP3 in DCs does not require additional signalling from ATP receptor P2X7, as macrophages require for their activation 81. Besides NLRP3/caspase 1-dependent activation of IL-1β secretion 82, the inflammasome-independent activation of pro-IL-1β has also been reported in DCs 83,84. Moreover, IL-1β was shown to be a direct target of HIF-1α 85, which we found upregulated in mature αKG-moDCs along with the intracellular levels of αKG. Therefore, it is possible that exogenous αKG directly increased the capacity of moDCs cells to produce IL-1β in an NLRP3-independent manner, while simultaneously impairing their differentiation and maturation capacity.

The differentiation and maturation of moDCs critically depend on the enzymes regulating their redox metabolism 25-27,29. Although αKG was shown to affect oxidative status in various cells 86,87, similar data for human DCs was missing. Here we found that αKG increased ROS early during their differentiation, which was followed by a strongly increased capacity of moDCs to express antioxidative enzymes, SOD and CAT, upon the stimulation with LPS/IFN-γ. ROS-induced transcription of these genes is regulated by Nrf2/KEAP-1 system 88, which also enhances HIF-1a expression 89. Accordingly, the increase in HIF-1α mRNA expression in immature αKG-moDCs could be linked to this phenomenon. LPS-induced ROS is a known mechanism of DCs maturation 90, inducing the increase in glycolysis rate 91 and destabilizing HIF-1α, which alone mediate pro-tolerogenic effects in DCs 26,92. Our findings suggested that αKG-moDCs are resistant to LPS/IFN-γ-induced degradation of HIF-1α. Previously, Hou et al. 93 showed that dimethyl-αKG can stabilize HIF-1α directly via inhibition of prolyl-4-hydroxylases and increase the anti-oxidative protection at the level of mitochondria and the glycolysis rate 94. Although we cannot exclude that similar, direct mechanisms were involved in HIF-1α stabilization by exogenous αKG in moDCs, our data indicated that without aKG-induced ROS, no HIF-1α stabilization occurs in moDCs after LPS/IFN-γ stimulation. Unlike the other studies 90,95, we did not observe a reduced intracellular ROS upon increased anti-oxidative protection, suggesting that αKG induced a constant oxidative stress in moDCs. In support of this, we identified a higher OXPHOS αKG-moDCs, which is the major source of ROS 96. The increased ROS can also trigger the AMPK-dependent inhibition of mTORC1 97, and the activation of Beclin-1 98, both of which increase autophagy 99. In contrast to our findings, Baracco *et al.* 100 showed that different αKG derivates inhibit autophagy depending on the nutrient conditions in cell culture media. Liu *et al.* 32 showed that dimethyl-αKG inhibited M1 polarisation in alveolar macrophages via inhibition of mTORC1 99, but autophagy was not analysed in these experiments. The increased autophagy in immature αKG-moDCs was most probably related to the induced expression of Beclin-1, a known autophagy inducer 98. Additionally, this phenomenon could be related to an increased level of pFoxO1 in αKG-moDCs. Namely, exogenous αKG may activate PI3K/Akt signalling via αKG-receptor GPR99 (OXGR1) 101, and this pathway leads to increased cytosolic FoxO1, which was found to directly induce autophagy in cancer cell lines by binding to Atg7 44. LPS was shown to reduce autophagy in moDCs under normoxic conditions 92, unlike IFN-γ which induces autophagy 102, which can explain the lack of significant change in the autophagy flux in LPS/IFN-γ-matured vs immature moDCs. However, LPS can also induce autophagy in moDCs under hypoxic conditions 92, which itself increases the autophagy in DCs via HIF-1α-mediated activation of class III PI3K/Vps34 and increased turnover of p62/SQSTM1 103. Therefore, it is possible that higher expression of HIF-1a, p62/SQSTM1 and increased pFoxO1/ total FoxO1 ratio contributed to the increased autophagy in mature αKG-moDCs. Thereby, we showed that these αKG-mediated processes highly depend on ROS and OXPHOS in moDCs. Additionally, we showed previously that the Beclin-1- and p62/SQSTM1-mediated induction of autophagy indeed leads to maturation block in moDCs 19, which is consistent with the phenomena observed in this study. However, additional studies are necessary to confirm this hypothesis and delineate the role of αKG in autophagy regulation.

Previous findings suggested that dexamethasone/VitD3-induced tolerogenic DCs display an impaired maturation capacity, and both increased OXPHOS and glycolysis 17, which is in line with our findings on αKG-DCs. Recently it was shown that Akt-mediated restriction of FoxO1 transcription in multiple myeloma cells increases their metabolic fitness characterized by increased glycolysis and OXPHOS 104. Moreover, the deficiency in FoxO1 results in lower expression of inflammatory response in macrophages 46, both of which align with our observations. Although OXPHOS and glycolysis are usually inversely regulated in macrophages 1, a recent study showed that TGF-β can uncouple these processes 105. These findings are consistent with our observation on increased TGF-β production by αKG-moDCs, irrespective of their maturation status. Moreover, both increased autophagy flux and increased HIF-1α expression were shown to characterise tolerogenic DCs 19,26,92. Opposite to our findings, Mangal *et al.* 34 reported that αKG-MP reduce OXPHOS and glycolysis in mouse bone marrow-derived DCs and potentiate the maturation of these cells in the presence of LPS, whereas soluble αKG only reduced glycolysis. However, it remained unclear how these changes affected the functional capacity of DCs since both αKG-MP- and soluble αKG-DCs reduced the proliferation of Th1, Th17, Th2 and Tregs in allogeneic MLRs 34. In subsequent work, these authors showed that αKG-MP increases the proportion of Th2 cells and decreases Th1 cells in ConA-stimulated mouse splenocytes, although the role of APCs remained unclear 106. Here we demonstrated that immature αKG-moDCs displayed a reduced capacity to induce Th1 cells, and an increased capacity for Th2 polarization, consistent with their weaker capacity to produce IL-12 and IP-10 107. Moreover, immature αKG-moDCs secreted higher levels of IL-10, which is known to decrease the Th1/Th2 ratio 67,71. Cheng et al. 108 showed previously that Kupffer cells increased the expression of IL-10 mRNA after the perfusion with dimethyl-αKG in a model of ischemia-reperfusion injury of the liver, which is in line with the M2 reprogramming of macrophages induced by dimethyl-αKG 31,32. However, this is the first time showing that a similar effect could be induced by exogenous αKG in human moDCs.

Besides increased IL-10, immature αKG-moDCs displayed an increased expression of IDO-1 which was directly involved in the induction of TGF-β-producing conventional Tregs. IDO-1 was shown to be upregulated via Gq-coupled receptors and the activation of PKC/PI3K/Akt pathway 109. OXGR1, expressed predominantly in monocytes and DCs 5, utilize the same Gq/11 pathway, so it might be involved in the induction of IDO-1 by αKG. However, unlike the succinate receptor GPR91 110, the role of GPR99 in immune modulation is poorly investigated, so further investigations are necessary. In line with our findings, both IDO-1 and IL-10 were shown critically involved in the induction of conventional Tregs by moDCs, via kynurenine-mediated activation of AhR and IL-10R, respectively 111,112. Interestingly, LPS/IFN-γ stimuli also upregulated IDO-1 expression in moDCs, suggesting the activation of negative regulatory mechanisms during the maturation as previously demonstrated 19,24. More importantly, IL-10- and IDO-1-mediated induction of conventional Tregs was not stably present in αKG-moDCs, as this capacity of αKG-moDCs was reduced after their maturation. Instead, mature αKG-moDCs displayed an increased capacity to induce Th17 cells. Conventional CD4^+^FoxP3^+^ Tregs and CD4^+^RORγt^+^ Th17 play essential roles in immune homeostasis and share the same signature transcription induced by TGF-β 113. Thereby, it was shown that IL-1β promotes Th17 differentiation by inducing the alternative splicing of FoxP3 114. These results are consistent with our observations that αKG-moDCs stably produced increased levels of TGF-β and increased production of IL-1β upon LPS/IFN-γ stimulation, most probably resulting in both an increase in the % of Th17 cells and the decrease of conventional Tregs. Additionally, mature αKG-moDCs displayed a stable production of IL-23, which was shown to contribute to IL-1β-mediated induction of Th17 cells, even in the absence of costimulation 115.

The key finding in this study was that αKG induced a strong expression of ILT-3, through which they induced non-conventional Tr1 and regulatory CD8^+^ T cells. ILT-3 expression in αKG-moDCs was dependent on functional OXPHOS, but independent of ROS, since in the presence of NAC, further potentiation of ILT3 by αKG was observed. The mechanism behind this phenomenon is still unclear and requires further investigation on redox-dependent and redox-independent pathways mediated by αKG. ILT-3 was previously described as the key regulator of IL-10-producing CD8 T and Tr1 cells 54,116, as we demonstrated previously 19,20,53. The non-conventional IL-10-producing T cells display stronger suppressive potential compared to conventional Tregs up to 50 times 20,117, and these cells can compose up to 30% of all solid tumour infiltrating lymphocytes 117. These results suggest that αKG may induce adverse tolerogenic effects in solid tumours via their actions on DCs and that previous claims on anti-tumour effects of αKG 8,118, should be taken with caution. Accordingly, we demonstrated previously that the blockage of ILT3 during the priming of T cells by tolerogenic DCs can also block the suppressive properties of IL-10-producing CD4 and CD8 T cells 19, which could be harnessed for the development of tumour therapy. On the other hand, the suppressive mechanisms mediated by Tr-1 cells can be beneficial in the treatment of autoimmunity and transplantation 119. However, αKG-moDCs-mediated induction of Th17 cells could also be observed as adverse in autoimmunity. Recent data suggested that the pathogenicity of Th17 cells highly depends on their metabolism, wherein targeting the glycolysis and OXPHOS in these cells may selectively affect the pathogenic subsets of Th17 cells 120. In this context, αKG might emerge as a desirable metabolic modulator of both DCs and T cells. Therefore, future investigation of immunomodulatory properties of αKG *in vitro* and *in vivo* models is necessary to resolve these issues and prevent its potentially adverse effects as a supplementation therapy.

## Conclusions

Here, we showed that exogenous non-esterified form of αKG displays potent anti-inflammatory activities in human model systems of APC/T cell interactions. For the first time, we showed that exogenous αKG impairs the differentiation and maturation of moDCs, mostly via alteration of their oxidative metabolism, HIF-1α stabilisation, and induction of autophagy. These changes induced an increased tolerogenic potential of αKG-moDCs and their capacity to induce different Treg subsets via IDO-1 and ILT-3. These effects of αKG are desirable for the generation of tolerogenic DCs intended for cell therapy. However, αKG-moDCs also displayed a preserved capacity to produce innate cytokines and induce Th17 cells after the maturation, which could be adverse for many chronic inflammatory diseases. Therefore, further investigations of immunomodulatory properties of exogenous αKG are necessary to better delineate its roles in immunometabolism and to avoid its potentially adverse effects during the application as a supplementation therapy.

## Supplementary Material

Supplementary materials and methods, figures.
