# Supplementary material for: Exogenous α-ketoglutarate Modulates Redox Metabolism and Functions of Human Dendritic Cells, Altering Their Capacity to Polarise T Cell Response
Source: Int J Biol Sci. 2024 Jan 20;20(3):1064–87. doi: 10.7150/ijbs.91109 (PMC10845299; doi:10.7150/ijbs.91109)
Supplement: Supplementary file 1 — Supplementary materials and methods, figures. [file ijbsv20p1064s1.pdf]

Supplementary Materials

Exogenous α-ketoglutarate Modulates Redox Metabolism and Functions of Human Dendritic Cells, Altering Their Capacity to Polarise T Cell Response

Marijana Milanović[1], Marina Bekić[2], Jelena Đokić[3], Dragana Vučević[1], Miodrag Čolić[2], Sergej Tomić[2]*

[1] Medical Faculty of the Military Medical Academy, University of Defense, Belgrade, Serbia

[2] Department for Immunology and Immunoparasitology, Institute for the Application of Nuclear Energy, University of Belgrade, Belgrade, Serbia

[3] Institute for Molecular Genetics and Genetical Engineering, University in Belgrade, Belgrade, Serbia

* **Correspondence:** sergej.tomic@inep.co.rs; Tel.: +381 11 2619-252

Materials and Methods

List of antibodies for Flow cytometry analysis

The following antibodies (clones) were used for staining moDCs and T cells in flow cytometry analysis, as indicated in Materials and Methods: IgG1 negative control-fluorescein isothiocyanate (FITC) (MCA928F), IgG1 negative control-phycoerythrin (PE) (MCA928PE) (all from Bio-Rad Laboratories, Hercules, California, United States); CD14-fluorescein isothiocyanate (FITC) (TUK4); CD1a- Peridinin-Chlorophyll-Protein (PerCP)/Cyanine (Cy) 5.5 (HI149), anti-HLA-DR-Allophycocyanin (APC)/Cy7 (L234), anti-CD209-APC (9E9A8), anti-CD83-FITC (HB15), CD86-PerCP Cy5.5 (IT2.2), CD11c Alexa Fluor 700 (Bu15), CD11b-PE-Cy7 (LM2), PD-L1-PE (29E.2A3), IL-33-biotin (poly5163), CCR7-PerCP-Cy5.5 (G043H7), IL-6-PE (MQ2-13A5), TNF-α-APC-Cy7, anti-IL-4-PerCP-Cy5.5 (MP4-25D2), anti-IFN-γ-FITC (4S.B3), IL-17A PerCP-Cy5.5 (BL168), CD8-PE-Cy7 (SK1), CD4-Alexa Fluor 700 (OKT4), anti-CD127-PE (A019D5), CD25-PerCP-Cy5.5 (M-A251), TGF-β-PE-Cy7 (TW4-6H10), anti-IL-10-APC (JES5-16E3), IgG1 negative control-PerCP-Cy5.5 (HTK888), PD1-APC-Cy7 (EH12.2H7), ILT3-PE-Cy7 (ZM4.1), CD73-FITC (AD2), anti-IL-10-PE (JES5-16E3), streptavidin-PerCP-Cy5.5, streptavidin APC, streptavidin PE-Cy7 (all from BioLegend); IgG1 negative control APC (MA5-18093), IL-17A-APC (eBio17B7), HIF-1α:biotin (H1alpha67) (all from Thermo Fisher Scientific), CD40-APC (5C3), anti-IL-12 (p40/p70)-PE (C11.5), anti-FoxP3-Alexa Fluor 488 (236A/E7), Fixable viability stain (FVS) 620, anti-RORγt-Alexa Fluor 488 (Q21-559) (all from BD Biosciences, San Diego, California, United States); anti-IL-1β-PE (rea1172), anti-GATA3-PE (REA174) (Miltenyi Biotec); anti-IDO-1-APC (700838), anti-TGF-β-PE (9016), anti-T-bet/TBX21-Alexa Fluor 647 (FAB53851R) (all from R&D Systems, Minnesota, United States).

Surface staining was performed in PBS/0.01% Na-azide for 30 min at 4°C. The cells were then washed in PBS and stained with FVS620 for 15 minutes, washed and then fixed using the fixation and permeabilization buffer (BioLegend) for the following incubation with antibodies for intracellular antigens. For each analysis, dead cells and doublets were excluded according to viability dye (FVS620) and forward scatter (FSC)-A/FSC-H, respectively. At least 5000 cells were gated according to their specific FSC-A/side-scatter (SSC)-A properties. Signal overlap between the fluorescent channels was compensated before each experiment by using compensation beads (ThermoFisher), and the non-specific fluorescence was determined by using appropriate isotype control antibodies. The samples were acquired on an LSR II flow cytometer with 8 colour channels (BD Biosciences) on the day of staining and analysed offline in FCS Express 7 software (De Novo software, California, United States).

List of primers for quantitative polymerase chain reaction

PCR primers used in the study were as follows: **GAPDH**_Foreward (F) GTGAAGGTCGGAGTCAACG, GAPDH_Reverse (R) TGAGGTCAATGAAGGGGTC; **SQSTM1**_F GCCAGAGGAACAGATGGAGT, SQSTM1_R TCCGATTCTG GCATCTGTAG; **BECN1**_F CTGGGACAACAAGTTTGACCAT, BECN1_R GCTCCTCAGAGTTAAACTGGGTT; **MAP1LC3B**_F TTCAGGTTCACAAAACCCGC, MAP1LC3B_R TCTCACACAGCCCGTTTACC; **AMBRA1**_F GGTGGGAGGAGAGGGGATAG, AMBRA1_R CGAGGGGCATGTCATCATTT; **CAT**_F AGTGATCGGGGGATTCCAGA, CAT_R AAGTCTCGCCGCATCTTCAA; **SOD1**_F ACAAAGATGGTGTGGCCGAT, SOD1_R AACGACTTCCAGCGTTTCCT; **HMOX1**_F GGGAAGATGCCATAGGCTCC, HMOX1_R CTCCCAGGGCCATGAACTTT; **HIF1A_F** GTCTGAGGGGACAGGAGGAT, HIF1A_R CTCCTCAGGTGGCTTGTCAG. All primers were designed with Primer Express v.3.0.1 (Applied Biosystems) and purchased from Thermo Fisher Scientific. GAPDH was performed on each experimental setup as an endogenous control.

Supplementary Figures

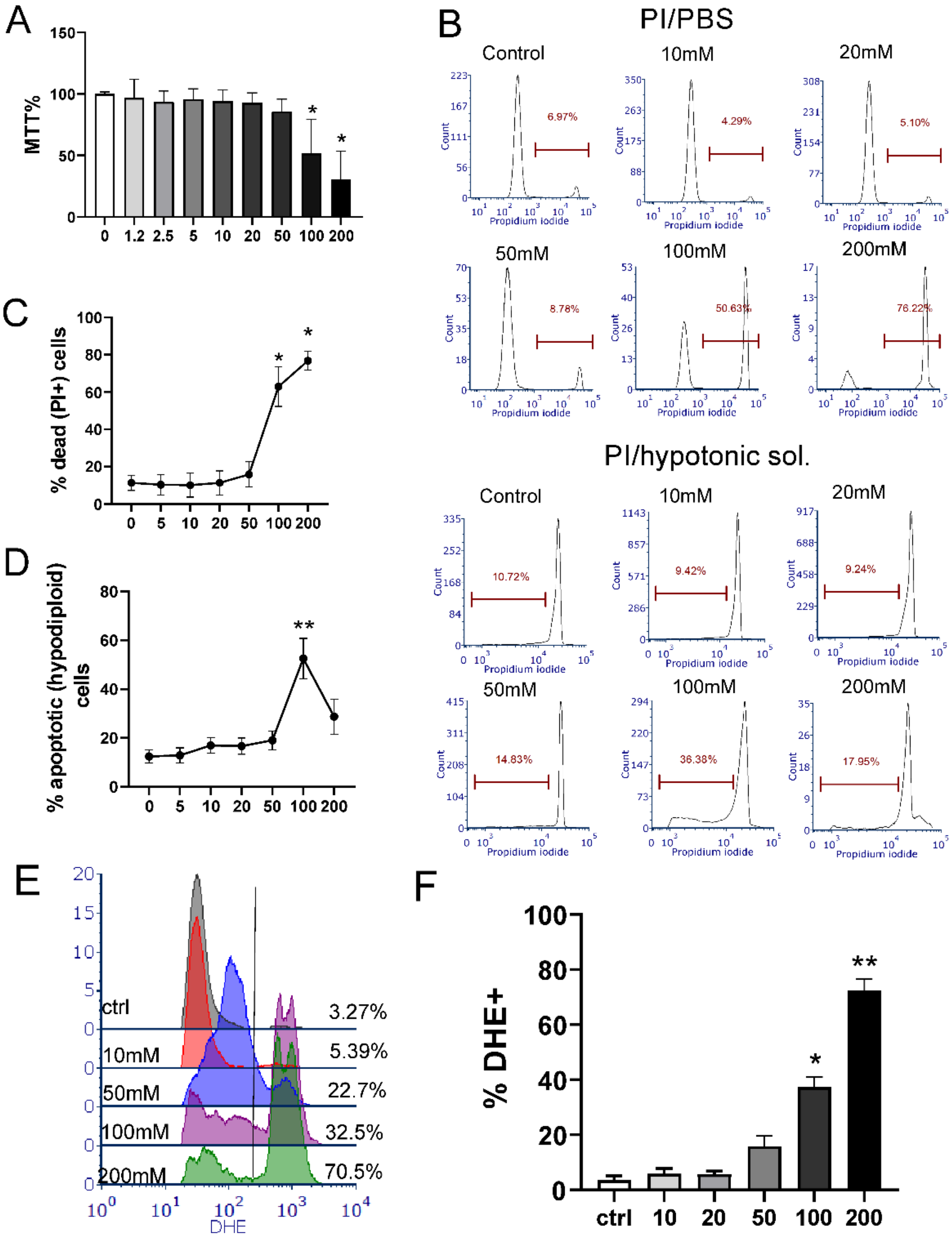

Supplement Figure 1. Cytotoxicity of exogenous α-ketoglutarate in human PBMC cultures. PBMCs (3x10^5 cells /well of 96-wells plate) were cultivated in the presence of αKG (1.2 - 200 mM) for 48h, and **A)** the relative metabolic activity (MTT %) measured in MTT assay is shown as mean MTT% ± SEM, from three different PBMC donors. **B)** The cell death of PBMCs in the cultures was measured by flow cytometry after staining the cells with PI in PBS (for necrosis analysis) or PI/hypotonic solution (for apoptosis analysis according to hypodiploid cells), as

indicated by representative histograms from one experiment. The summarized data on % of PI+ (necrotic cells) **(C),** and apoptotic (hypodiploid) cells **(D)** are shown as mean % ± SEM from three independent experiments**. E)** A representative analysis of ROS levels in PBMCs, as determined by DHE staining after the cultures with indicated doses of αKG, is shown, and F) The summarised data from three independent experiments are shown as mean ROS% ± SEM. **(A, C, D, F)** *$p<0.05$, **$p<0.01$ compared to control (ctrl) (RM-ANOVA, Dunnett post-test).

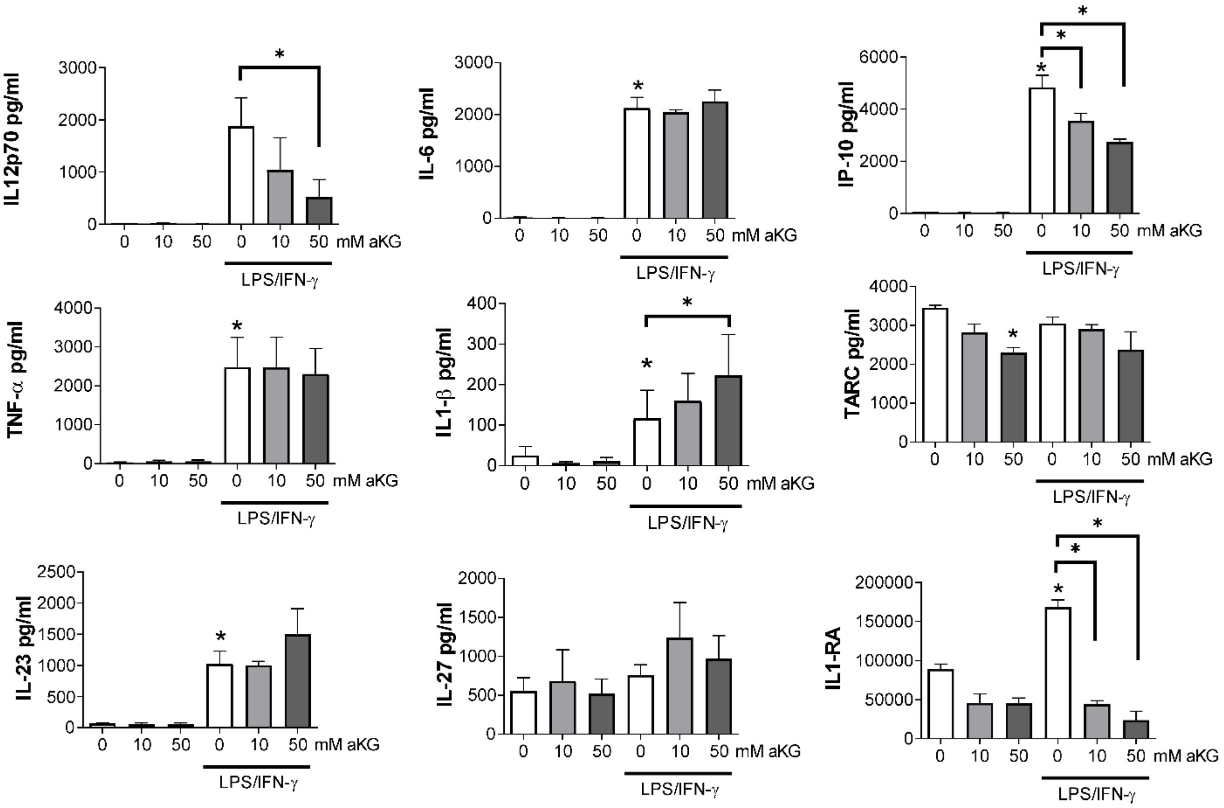

Supplement Figure 2. Cytokine levels in moDCs culture supernatants after the stimulation with LPS/IFN-γ. MoDCs were generated with GM-CSF and IL-4, either in the presence of αKG (10 mM or 50 mM) or its absence for 4 days, followed by the stimulation with LPS/IFN-γ, or not, for the next 16-18h. The cytokine levels were determined from moDCs culture supernatants on day 5 by flow cytometry or ELISA for IL-27, and the results are shown as mean pg/ml ± SEM, from 3 experiments carried out with different donors.

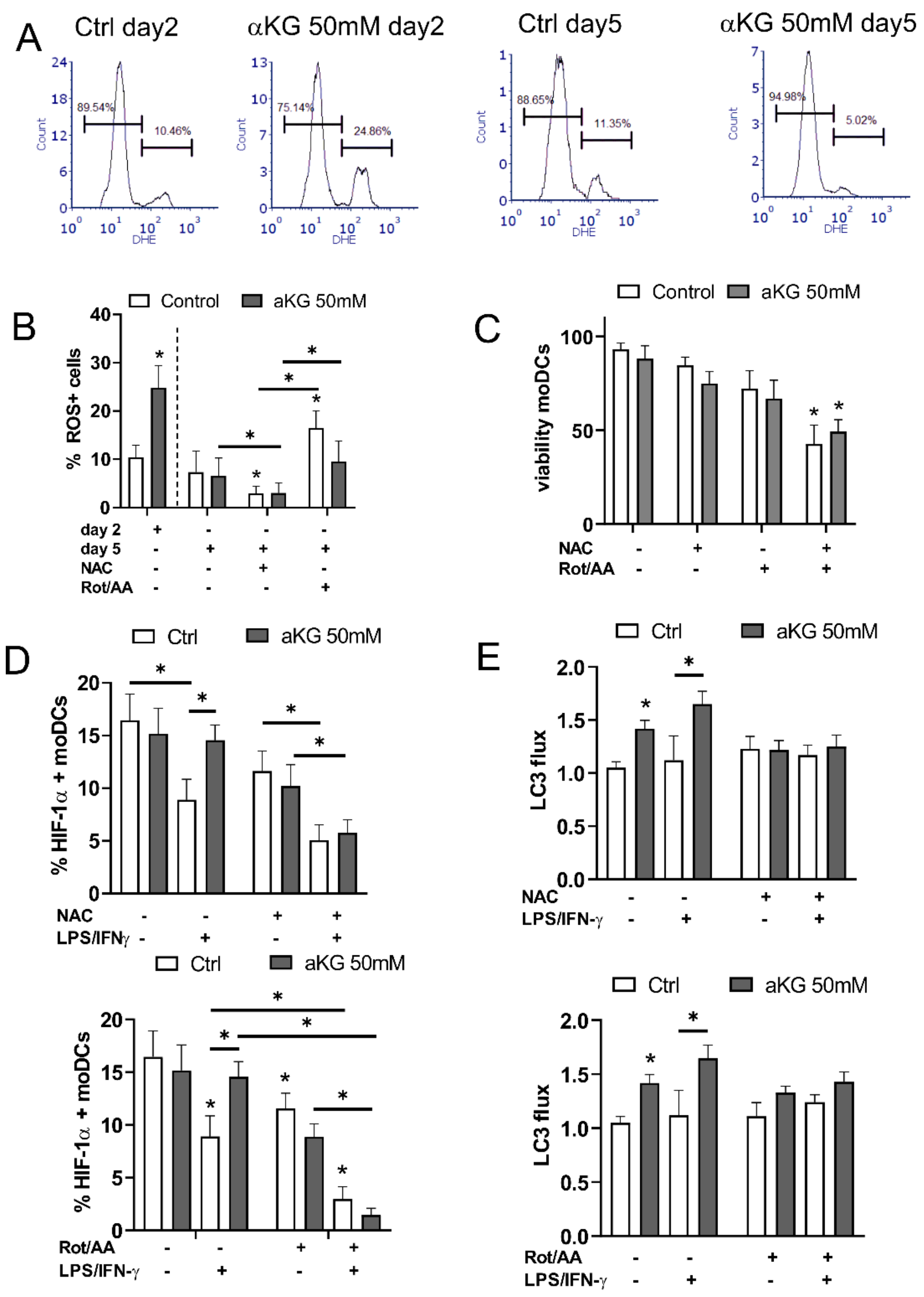

Supplement Figure 3. Regulation of ROS, HIF-1α and autophagy in αKG-moDCs. MoDCs were generated with GM-CSF and IL-4, either in the presence of αKG (50 mM) or its absence for 4 days, and then stimulated with LPS/IFN-γ or not for the next 16-18h. Some cultures were treated with NAC (10 mM), Rot(0.5 μM)/AA(0.5 μM) or their combination, starting on day 2. **A)** Representative histograms are shown, of intracellular ROS measured in moDCs on day 2 or day 5 of the cultures, by DHE staining, and **B)** the summarized data is shown as mean ± SEM (n=3 different moDC donors). **C)** Viability of moDCs, according to the analysis of moDCs on day 4 of the cultures (i.e. before LPS/IFN-γ stimulation) by Muse Count/Viability assay, is shown as mean

% ± SEM (n=3). **D)** The expression of HIF-1α protein levels was measured on Day 5 by flow cytometry, and the summarized results obtained in NAC-treated, Rot/AA-treated, and non-treated moDCs, are shown as % of HIF-1α$^+$ cells ± SEM (n=3), as identified according to isotype control staining. **E)** The summarized data on autophagy flux measurements, representing the ratio of membranous LC3-II expression in Bafilomycin-treated (Baf) and non-treated (ctrl) moDCs, carried out in NAC-treated, Rot/AA-treated, and non-treated moDCs are shown as mean flux ± SEM (n=3). **D, E)** moDCs differentiated without NAC or Rot/AA were shown twice for easier comparison. *p<0.05 αKG-treated vs corresponding non-treated moDCs, or as indicated (RM-ANOVA, Dunnett's post-test).

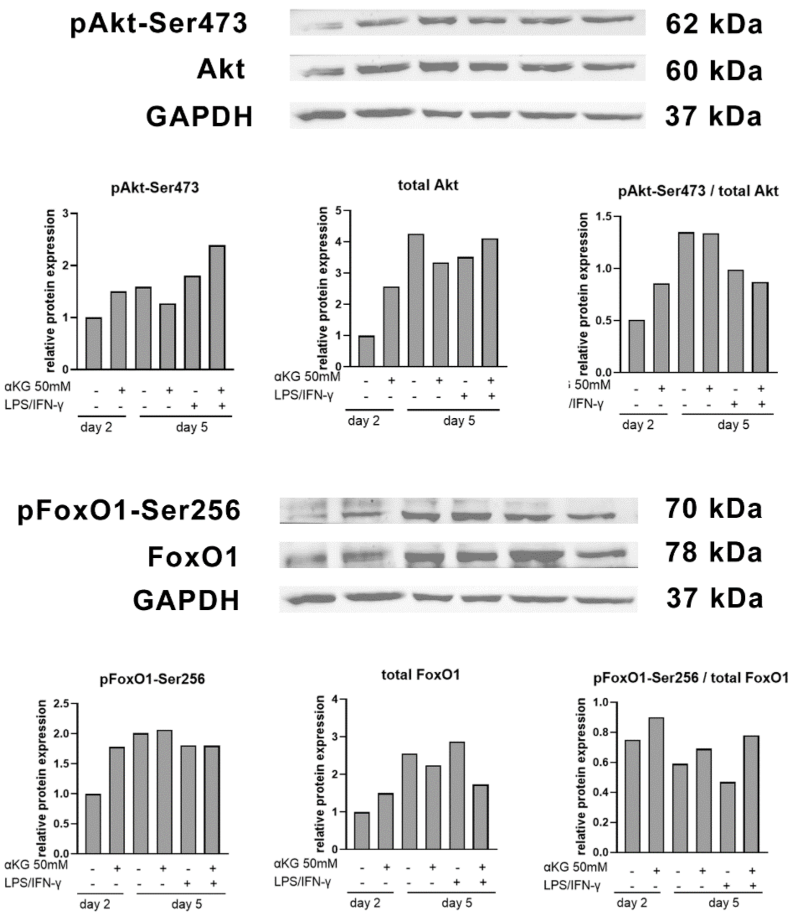

Supplement Figure 4. Protein expression and phosphorylation of protein kinase B (Akt) and Forkhead box protein O1 (FOXO1) signalling molecules in moDCs. Protein expression of Akt, phosphorylated Akt at Ser473, total FOXO1, and phosphorylated FOXO1 at Ser256 were determined from moDC lysates and representative immunoblots are shown. The relative expression of the proteins was calculated according to GAPDH expression as a loading control. The results from one experiment, out of two with similar results, are shown below the immunoblots.

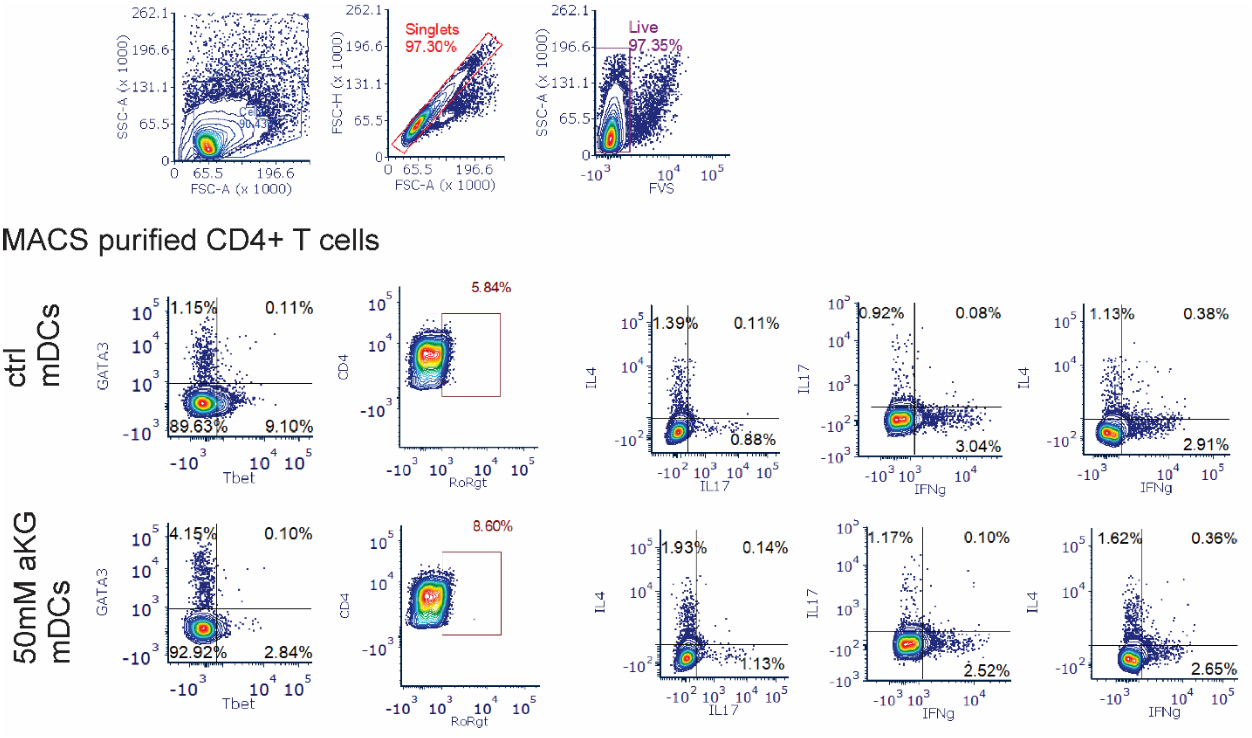

Supplement Figure 5. The expression of transcription factors and cytokines in CD4^{+}T cells co-cultivated with mature moDCs or αKG-moDCs. The expression of T-*bet*, GATA-3, ROR-*γt* and co-expression of IL-4, IL-17 and IFN-γ was analysed in CD4+T cells co-cultivated with mature moDCs or αKG-moDCs. CD4^{+} T cells were first MACS purified from allogeneic PBMC and then co-cultivated with mature moDCs for 5 days, and PMA/ Ca ionophore/ monensin for the last 4h. The gating strategy (top row) shows debris and singlets exclusions, and dead cells exclusion according to the FVS staining. One experiment is shown, out of two with similar results.

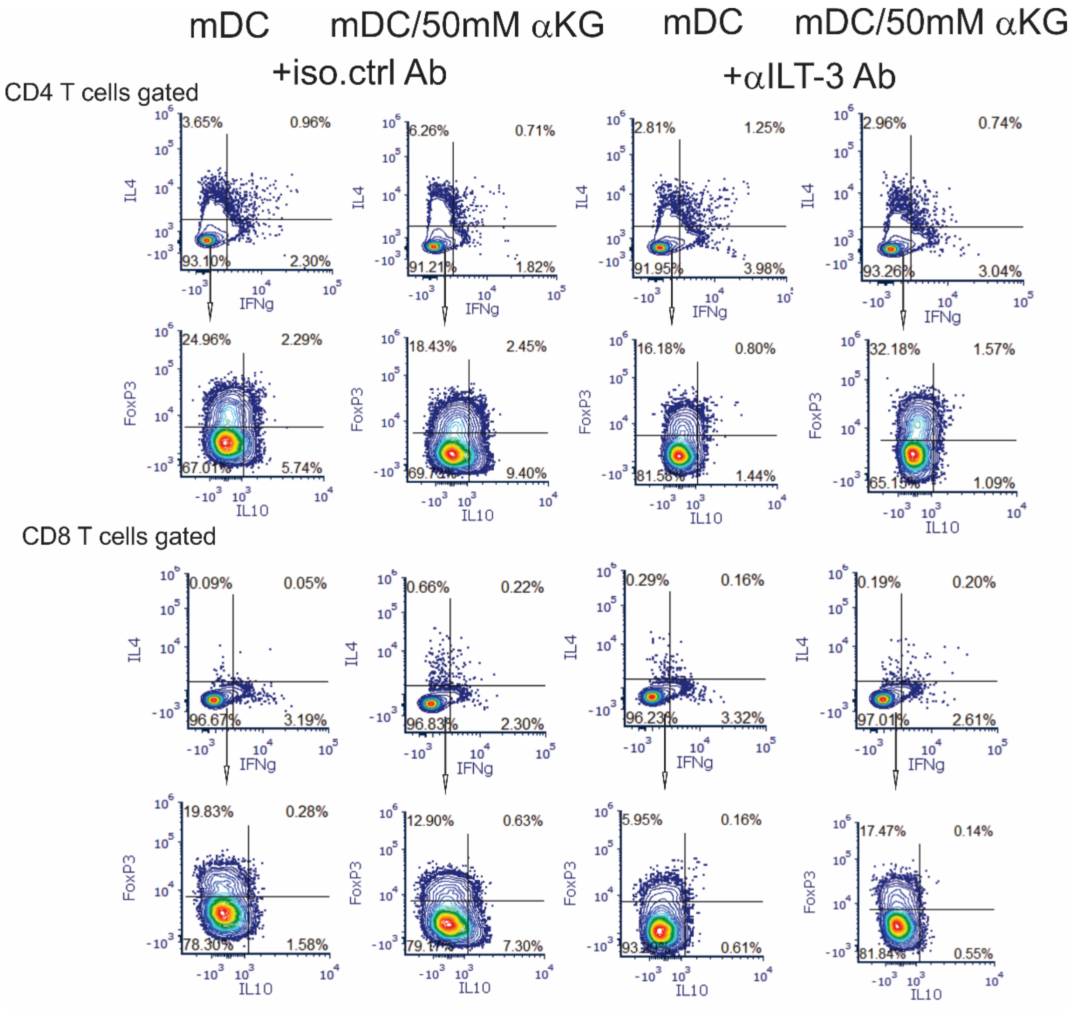

Supplement Figure 6. Analysis of Tr1 and suppressor CD8+T cells in co-cultures with moDCs. MoDCs/ T cell co-cultures were carried out at 1:50 (moDC: T cell ratio) in the presence of IL-2 for 6 days and either in the presence of isotype control Ab or anti-ILT-3 Ab. Tr-1 cells were gated within $CD4^+$ T cells and then within IFN-γ^- IL-4^- and $FoxP3^-$ $IL10^+$ cells, whereas IL-10^+CD8^+ T cells were gated likewise, but within $CD8^+$ T cells. A representative experiment performed with mature moDCs is shown.
